# A Multi-Site Measurement System with Photoplethysmographic and Microwave Sensors for Synchronous and Continuous Cardiovascular Signal Acquisition

**DOI:** 10.3390/s26196311

**Published:** 2026-10-06

**Authors:** Adrian Nigot, Weronika Giblewska, Tadeusz Sondej, Zenon Szczepaniak

**Affiliations:** 1 Faculty of Electronics, Military University of Technology, 00-908 Warsaw, Poland; tadeusz.sondej@wat.edu.pl (T.S.); zenon.szczepaniak@wat.edu.pl (Z.S.); 2 MediSensonic SA, 52-234 Wrocław, Poland; weronika.giblewska@gmail.com

**Keywords:** biomedical signals, multimodal and multi-site measurement system, non-contact pulse wave measurements

## Abstract

**Highlights:**

**What are the main findings?**

- A multimodal and synchronous platform combining PPG, multispectral PPG, microwave sensing and ECG was successfully developed and evaluated for multi-site cardiovascular signal acquisition.
- Pulse waveform morphology and signal quality depend on both sensing technology and measurement location, with microwave signals showing particularly pronounced inter-subject variability.

**What are the implications of the main findings?**

- Combining PPG and microwave sensing or other modalities enables synchronized multimodal acquisition of cardiovascular-related signals and provide complementary information for further investigation under controlled conditions.
- The observed site- and subject-dependent differences highlight the importance of sensor placement and individualized signal interpretation in future cardiovascular monitoring and parameter-estimation methods.

**Abstract:**

The dynamic development of non-invasive medical diagnostics and continuous health monitoring using biomedical signals in recent years has been one of the main research directions in biomedical electronics. This paper presents a developed multimodal, multi-site, and synchronous measurement system designed for continuous cardiovascular signal acquisition. The system integrates reflective and transmissive photoplethysmographic (PPG) sensors and active microwave (MW) sensors operating at a frequency of 4.9 GHz. One of the reflective PPG sensors enables multispectral (MWPPG) recording for six different light wavelengths, ranging from blue (465 nm) to infrared (940 nm). The system was evaluated in a trial involving a group of 32 volunteers. During our research, the PPG sensors were placed on the fingers of the left hand, wrist, and forehead, while the MW sensors were placed on the thumb, wrist, biceps, and chest. In addition, ECG signals and accelerometer signals are recorded synchronously. The averaged pulses of the individual sensors were appointed to compare their shape and quality depending on the technology and location of the measurement. The PPG signals exhibited higher signal quality, with mean SNR values ranging from 8.66 ± 2.17 to 12.61 ± 3.98 dB, compared with 5.91 ± 0.98 to 7.95 ± 1.26 dB for the MW signals. Similarly, the pulse-detection F1-score ranged from 0.34 ± 0.22 to 0.74 ± 0.17 for PPG and from 0.33 ± 0.07 to 0.43 ± 0.15 for MW sensors. Significant differences in the shape of the pulses from the MW sensors were also demonstrated between individual participants. Furthermore, the paper presents examples of recorded signals and discusses the advantages and limitations of various sensor technologies and their locations, while also indicating potential directions for further research.

## 1. Introduction

The rapid growth of the biomedical field has opened up new possibilities for continuous non-invasive health monitoring and support for early medical diagnosis [1,2,3,4]. Modern measurement systems enable long-term recording of biomedical signals under conditions similar to natural functioning [1,3,4,5]. Unlike traditional diagnostic methods, continuous measurements allow the observation of dynamic changes and the detection of subtle deviations from the norm that may go unnoticed during one-time tests [2,3,6]. Biomedical signals are a valuable source of information on the state of the body’s systems [2,4]. However, their acquisition requires the use of appropriately designed measurement systems that ensure high signal quality, resistance to interference, and user comfort [3,4,7]. A significant challenge in designing such systems is achieving a balance between precision of measurement, operational stability, and energy efficiency [1,3,7]. The recorded biomedical signals form the basis for the determination of a range of important physiological and diagnostic parameters, which can be used in both health monitoring and diagnostic processes [2,4,5]. Based on bioelectrical signals, such as the electrocardiogram (ECG) or electromyogram (EMG), it is possible to determine, among other things, heart rate (HR) and heart rate variability (HRV), detect cardiac arrhythmias, and assess muscle activity [4,8,9]. In turn, analysis of optical signals such as photoplethysmography (PPG) allows the determination of, among other things, pulse rate (PR), pulse rate variability (PRV) [10,11], blood oxygen saturation (SpO_2_), pulse wave propagation time, and, indirectly, blood pressure (BP) [2,6,12]. Additionally, biomedical signals related to physical and biomechanical activity, obtained from inertial sensors, allow for the assessment of physical activity levels, gait analysis, body posture, and the identification of episodes of immobility or falls [3,4,5]. Combined with physiological data, this enables complete monitoring of user health and assessment of body response to exercise, stress, or environmental changes. Signal processing, machine learning (ML) and deep learning (DL) algorithms are also being increasingly used; based on a set of parameters derived from biomedical signals, they allow the prediction of health parameters and the early detection of pathological conditions [2,3,5].

The pulse wave signal is one of the primary sources of information used in research on the analysis of cardiovascular function. Its recording can be performed using various measurement techniques and sensor types, which differ in their operating principles, sensitivity, and range of applications. One of the most commonly used methods is PPG, which uses optical analysis of changes in blood volume within blood vessels based on changes in light absorption and reflection. Alternatively, mechanical sensors—such as piezoelectric, piezoresistive, or strain gauge sensors—are used to record slight changes in pressure or skin surface deformations caused by the propagation of the pulse wave in the arteries [13,14]. Some systems also use capacitive or microelectromechanical (MEMS) sensors, which allow the detection of subtle vibrations associated with cardiovascular activity [15]. Non-contact techniques using electromagnetic waves are also attracting increasing interest. Examples include microwave (MW) and radar sensors, which enable the detection of pulse waves by analyzing changes in the phase or frequency of the reflected signal [16,17]. Other methods are also being developed, such as ultrasonic systems, interferometric sensors, biomagnetic field sensors, and video imaging techniques [18,19]. The selection of the appropriate technology depends, e.g., on the precision of the measurement required, the resistance to interference, and the measurement conditions.

This paper presents a multimodal and multi-site measurement system developed for various advanced analyzes of biomedical signals. The system performs synchronous measurement of biomedical signals—ECG, MW, and PPG signal at six different light wavelengths ranging from blue (465 nm) to infrared (940 nm). This multimodal data acquisition creates a rich database of measurements with significant research potential. Data obtained from such a system can serve as the basis for developing advanced signal analysis algorithms and ML methods, enabling the identification of more complex diagnostic indicators, e.g., related to the development of cardiovascular diseases (CVDs) [20,21,22]. The use of sensors located at various locations on the body enables a comparative analysis of individual measurement techniques depending on the type of sensor and its location. It also allows for the assessment of the impact of the measurement location on the characteristics of the recorded biomedical signals and expands the possibilities of hemodynamic and physiological analysis based on the collected data. Synchronous signal recording is of key importance in this type of research, as it ensures temporal consistency between biomedical signals recorded. This enables a proper analysis of the relationships between them, including the assessment of pulse wave propagation time [6,20,23].

The main contributions of this paper are as follows:

- Multimodal, multi-site, and synchronous measurement system designed for continuous cardiovascular signal acquisition;
- Development of a novel MW sensor for contactless physiological measurement;
- Implementation of a multispectral PPG (MWPPG) sensor;
- Multi-site measurements that allow for the assessment of signal quality based on location;
- In-depth analysis of the MW pulses morphology.

Moreover, this paper will help researchers and practitioners in developing advanced multimodal cardiovascular signal acquisition systems, with particular focus on MW sensors. It highlights the importance of sensor placement, which has a decisive impact on signal quality, as well as the need for subject- and location-dependent analysis of MW pulse morphology due to significant inter-individual variability.

Most existing cardiovascular monitoring systems are based on a limited number of sensing modalities and measurement locations. The proposed platform differs from previously reported multimodal cardiovascular monitoring systems by integrating synchronized ECG, multispectral PPG (MWPPG), multi-site PPG, and multi-site microwave (MW) sensing within a single hardware and software framework, enabling simultaneous and time-synchronized acquisition of complementary cardiovascular signals across optical, electrical, and radar-based modalities. The integration of MWPPG and MW sensing combines two complementary measurement modalities within a single synchronized acquisition framework. MWPPG enables optical assessment of peripheral vascular responses at different wavelengths, while MW sensing provides information from deeper tissue layers. Although both MWPPG and MW sensing are sensitive to cardiovascular pulsations and blood flow dynamics, they rely on different physical principles and probe different tissue depths. In addition, PPG measurements require direct optical contact with the skin and are more sensitive to local perfusion conditions, whereas MW sensing is less affected by skin pigmentation or ambient lighting conditions. As a result, their combined use provides a more comprehensive characterization of pulse wave propagation, vascular function, and cardiovascular dynamics than either modality alone. Furthermore, the synchronized acquisition of both signals facilitates direct comparison of measurements obtained using different sensing principles, enabling more reliable evaluation of cardiovascular signal acquisition techniques. Consequently, the integration of these modalities may increase the diversity and complementarity of the acquired cardiovascular information, potentially improving the robustness and stability of multimodal analysis. Detailed comparison with previously reported systems is provided in Section 4.7.

The rest of the paper is organized as follows. Section 2 provides a detailed description of the measurement system, individual sensors, data synchronization method, characteristics of recorded signals, computer software, and signal processing methods. The results of the work, in the form of the developed measurement system, sample recordings, and averaged pulses, are presented in Section 3. Section 4 (Discussion) discusses the advantages and limitations of the system, the sensor technologies used, the analysis of averaged pulses, and a comparison of the system with other similar solutions. Section 5 describes capabilities of the proposed measurement system. The conclusion (Section 6) summarizes the results of the work and identifies future directions for research and development.

## 2. Materials and Methods

### 2.1. Characteristics of the Measurement System

The designed measurement system (MS) is intended for multimodal, multi-site, and synchronous data acquisition for research related to biomedical signals recording, particularly in the cardiovascular system. The MS was designed to be portable and capable of recording data even during daily activities. Its main task is to measure and record signals from various sources (sensors) and to provide real-time (online) visualization of these signals. A block diagram of the designed MS is presented in Figure 1.

The main component of the MS is the Personal Sensor Hub (PSH) module, which acts as a signal integrator (hub) for all other sensors placed on the body. Its functions include powering the sensors, synchronous data acquisition from the sensors, saving data to an Secure Digital (SD) card, and communication with the computer. A Universal Serial Bus (USB) interface with galvanic isolation and specialized data visualization software is used for communication with the PSH. But this is an optional functionality because PSH can work autonomously.

The sensors communicate with the PSH module via a wired connection using a Universal Asynchronous Receiver–Transmitter Interface (UART). In addition, all sensors are equipped with an accelerometer to measure physical activity. The recorded data from the measurement system are read offline after the recording is complete. The recording time can exceed 24 h. The developed measurement system features sensors located at various points in the body. This makes it possible to conduct various comparative trials, e.g., regarding cuffless and continuous BP estimation using, among other things, Pulse Arrival Time (PAT) and Pulse Transit Time (PTT) or the use of different LED wavelengths. Figure 2 shows the arrangement of individual MS modules and sensors.

Our MS consists of the following components:

- PSH—main module;
- S1—PPG reflective sensor on the left wrist with ECG measurement;
- S2—PPG transmission sensor on the index finger of the left hand;
- S3—MWPPG reflective sensor on the middle finger of the left hand;
- S4—PPG reflective sensor on the forehead;
- S5—MW sensor on the chest (above the heart);
- S6—MW sensor on the left wrist;
- S7—MW sensor on the left bicep;
- S8—MW sensor on the left thumb;
- Mobile computer (enabling live signal viewing).

During the development of the system, attention was paid to the ease of sensor placement and patient comfort after application. Two PPG sensors were placed on the fingers of the left hand, performing measurements using both the transmissive (S2) and reflective (S3) methods. This decision was made because the fingers have excellent blood flow, enabling the acquisition of high-quality PPG signals and the ability to perform a further detailed analysis. An MW sensor (S8) was placed on the thumb. The PPG sensor on the wrist (S1) was positioned there because it is an ideal location to use during daily activities. An MW sensor (S6) was placed on the lower part of the wrist (above the radial artery). S4 was placed on the forehead because the blood flow is very good there and its location is comfortable enough that it can also be used in commercial applications. The chest MW sensor (S5) was used to record heart activity. Another potentially good location, due to access to the brachial artery, is the inner part of the biceps, where the MW sensor (S7) was placed. The ECG electrodes were placed in the standard manner on the chest electrode (RA (−) under the right clavicle, LA (+) under the left clavicle, and REF electrode on the right side of the torso).

### 2.2. Construction of PPG Sensors

PPG sensors consist of two printed circuit boards (PCB)—a main PCB and a measurement PCB. The main PCB contains a microcontroller (MCU) and is responsible, e.g., for power supply, communication with the PSH, and measurement of physical activity. The measurement PCB, in turn, contains circuits for acquiring PPG and ECG signals. This architecture allows for an easy replacement of the measurement section without the need to redesign the entire sensor. PPG sensors are based on an MCU from the STM32L552 family (STMicroelectronics, Plan-les-Ouates, Switzerland) and communicate with the PSH via a UART interface. The AFE4404 chip (Texas Instruments Incorporated, Dallas, TX, USA) is used for the measurement of the PPG signals in sensors S1 and S4 and the AFE4490 chip (Texas Instruments Incorporated, Dallas, TX, USA) in sensor S2, while the ADS1292 (Texas Instruments Incorporated, Dallas, TX, USA) chip is used for the measurement of the ECG signal. In addition, each sensor is equipped with an ADXL362 accelerometer (Analog Devices, Inc., Wilmington, MA, USA) to measure physical activity. The accelerometer is connected to the MCU via an Serial Peripheral Interface (SPI). The block diagrams of the individual PPG sensors are shown in Figure 3.

In S1 and S4, the SFH7060 optical module (OSRAM Opto Semiconductors GmbH, Regensburg, Germany) was used, which features three LEDs: green (530 nm), red (660 nm), and infrared (950 nm), as well as a photodiode. Two SFH7060 modules were used for measurements, placed side by side at a distance of 5 mm (Figure 4d). This enables parallel measurement of a larger number of PPG signals, thereby increasing the likelihood of capturing the desired signal. A Nellcor DS-100A probe (Covidien llc, Mansfield, MA, USA) was used as the PPG transmission sensor on the index finger (S2). Both PCBs of the example PPG sensor are shown in Figure 4.

### 2.3. Construction of MWPPG Sensors

The construction of the MWPPG (S3) sensor is the same as that of other PPG sensors. The main difference is the use of the modern AFE4950 chip (Texas Instruments Incorporated, Dallas, TX, USA) for PPG measurement, which supports synchronous measurement for 8 different LEDs, enabling multispectral PPG measurement. The S3 sensor has its own array of LEDs arranged side-by-side in a column and 5 mm away from the photodiode (Figure 5). The following LEDs were used: infrared (940 nm); red (624 nm); orange (601 nm); yellow (590 nm); green (515 nm); blue (465 nm); and an SFH2201 photodiode (ams-OSRAM AG, Premstaetten, Austria).

### 2.4. Construction of MW Sensors

MW sensors also consist of two connected PCBs, similar to PPG sensors. Their block diagram is shown in Figure 6.

The measuring head of the MW sensors (S5–S8) consists of two functional blocks: a microwave signal generation circuit and analog front-end (AFE). The generator circuit produces a radio frequency (RF) signal with a carrier frequency of 4.9 GHz, while the AFE is responsible for the reception and conditioning of the reflected signal from the tissue before ADC. The measurement antenna is designed as an asymmetric microstrip line (MSL) and is directly integrated with the microwave circuit. The design used (Figure 7) enables a compact measurement head and stable signal propagation conditions.

The operating principle of the MW sensor is based on the emission of a continuous microwave signal that propagates toward the biological tissue via the electromagnetic wave applicator. The electromagnetic wave penetrates the skin and subcutaneous tissue, where it interacts with vascular structures and moving blood. Part of the transmitted energy is reflected back toward the sensor, while the motion of blood and tissue caused by cardiovascular activity introduces phase and frequency variations in the reflected signal. As a result of the Doppler effect, these variations are related to periodic changes in blood flow and tissue displacement associated with the cardiac cycle. After amplification and conditioning in the analog front-end, the resulting signal can be used to extract pulse-related information. The carrier frequency of 4.9 GHz was selected as a compromise between tissue penetration depth, sensitivity to small tissue displacements, electromagnetic wave applicator dimensions, and hardware complexity. At this frequency, the estimated penetration depth in biological tissue is approximately 9.6 mm, which is sufficient for monitoring pulse-related activity at the selected measurement locations, including the thumb, wrist, biceps, and chest region. These anatomical sites contain superficial arteries or tissue structures influenced by cardiovascular activity that can be effectively observed within the achievable penetration depth. At the same time, the selected frequency maintains sufficient sensitivity to detect small pulsatile movements while enabling compact sensor implementation.

Compared with higher-frequency radar-based biomedical sensing systems operating at frequencies such as 77 GHz [24], the selected operating frequency of 4.9 GHz provides greater penetration depth into biological tissues and lower propagation losses. However, these advantages are achieved at the expense of lower spatial resolution and reduced sensitivity to very small localized movements. In addition, unlike frequency-modulated continuous-wave (FMCW) radar systems, the proposed continuous-wave Doppler approach does not provide direct range information. Nevertheless, its simpler hardware architecture and lower computational complexity make it suitable for continuous multimodal cardiovascular monitoring.

Since the MW sensor is a device that emits electromagnetic waves, it must comply with specific guidelines regarding the power absorbed by human tissue. To this end, an analysis of tissue exposure to the electromagnetic field was conducted. The aforementioned guidelines are cited in 1999/519/EC and IEEE C95.1-2019. The specific absorption rate (SAR) value of the MW system was simulated using CST Studio Suite (2024) [25]. The following assumptions were made for the simulation: SAR calculated for a tissue mass of 10 g according to the IEEE/IEC 62704-1 method; the input power in the simulator (default value) is 13.28 mW. The simulation results obtained for several different tissue thickness configurations are as follows:

- Total SAR = 0.002–0.005 W/kg.
- Maximum SAR = 0.041–0.117 W/kg.

SAR calculations assuming continuous operation and the maximum obtained value yield the following results:

- Total SAR = 0.005 W/kg.
- Maximum SAR = 0.117 W/kg.

For the intended use of the sensor as an application on the skin of the hand, the SAR limit is 4 W/kg. This means that the total SAR value is approximately 800 times lower than the limit value and the maximum SAR value is approximately 34 times lower than the limit value.

### 2.5. Method of Synchronization and Data Acquisition from Sensors

The synchronization of signal acquisition from each sensors of the measurement system was implemented using the interrupt system of an ARM Cortex-based MCU, the Nested Vectored Interrupt Controller (NVIC) and event handling prioritization. Communication between the PSH and the sensors is carried out using a UART interface, which enables the transmission of measurement data and synchronization signals between devices. To ensure the temporal consistency of the recorded data, a polling mechanism was also used for each sensors with a fixed response time (no longer than 1 ms). This approach allows maintaining a controlled temporal structure of data acquisition and minimizing delays in the MS, which is particularly important in the case of synchronous recording of multiple biomedical signals.

The MS performs synchronous acquisition of signals from various sources. Sensors S1 and S4 record 2 PPG channels from an infrared LED, 2 PPG channels from a red LED, 2 PPG channels from a green LED and 3 axes (XYZ) from an accelerometer. In addition, S1 also records an ECG signal (1 lead). S2 records PPG signals from infrared and red LEDs. The S3 multispectral sensor records one signal each from an infrared, red, green, orange, yellow, and blue LED, as well as 3 axes (XYZ) from the accelerometer. In the case of MW sensors (S5–S8), each records a single MW signal and 3 axes (XYZ) from the accelerometer. PPG and ECG signals are recorded at a sampling frequency of *f_s_* = 250 Hz, MW signals at *f_s_* = 1 kHz, and accelerometer data at *f_s_* = 100 Hz.

### 2.6. Computer Control Software

A dedicated application called RTSignalPlot (version 1.0) was developed for the online visualization of data from the MS (the main application window is shown in Figure 8). Communication between the application and the PSH module is carried out via a wired USB interface (using a UART/USB converter). The application enables the reception and visualization of measurement signals in real time. The software was developed in the Matlab (R2025a) environment using the AppDesigner tool (R2025a). The application allows for simultaneous viewing of multiple signals, their filtering using configurable digital filters, and real-time Fast Fourier Transform (FFT) spectral analysis. In addition, a feature to record measurement data, receive diagnostic messages sent from the measurement device, and send control messages has been implemented. The use of this software is optional, but is useful when verifying the correct placement of sensors.

### 2.7. Signal Processing

The purpose of our analyzing the recorded signals from the developed MS was to determine the averaged ECG, PPG, and MW signals from individual sensors (locations). To this end, an algorithm was developed in the Matlab environment.

A cascade of Infinite Impulse Response (IIR) filters was applied to the ECG signal, consisting of a 3rd-order Chebyshev type II high-pass filter (0.2 Hz, 40 dB stopband attenuation) followed by a 5th-order Chebyshev type II low-pass filter (25 Hz, ≥30 dB attenuation). The high-pass filter was used to remove baseline wander, while the low-pass filter suppressed high-frequency noise. Additionally, a 4th-order Chebyshev type II notch filter (49–51 Hz, 30 dB attenuation) was used to suppress power-line interference [26].

PPG signals were filtered using a cascade of two IIR filters: a 3rd-order Chebyshev type II high-pass filter (0.2 Hz, 40 dB attenuation) and a 5th-order Chebyshev type II low-pass filter (10 Hz, ≥30 dB attenuation). These filters were used to remove low-frequency baseline fluctuations and high-frequency noise while preserving pulse wave morphology.

MW signals were processed using a cascade of Chebyshev type II filters, including a 3rd-order high-pass filter (0.2 Hz, 40 dB stopband attenuation) and an 8th-order low-pass filter (15 Hz, 30 dB attenuation). The selected passband was chosen to preserve pulse-related components while reducing low-frequency drift and high-frequency interference. To compensate for nonlinear phase distortion introduced by IIR filtering, a zero-phase filtering algorithm was applied [27].

The well-known Pan–Tompkins algorithm was used to detect R-wave peaks in the ECG signal [28,29]. In the case of the PPG signal, there are many different fiducial points [30]. The most commonly used points to determine the onset of pulse wave are: the PPG signal maximum; the maximum of the I or II derivative; the minimum value marking the start of the PPG signal pulse; or the foot point, which can be defined as the intersection of two tangents, such as those occurring at the maximum of the I derivative and at the minimum value of the PPG pulse [31,32]. The algorithm developed implements the determination of the onset of pulse wave on the basis of the minimum value (local minimum). Based on the fiducial points determined for the ECG and PPG, individual pulses were extracted from the filtered signals.

For PPG and MW signals, individual pulses were defined between consecutive minima (from *t_N_* to *t_N_*_+1_). For ECG signals, segmentation was performed based on adjacent R-peaks, where each segment spans from (*t_N_*_−1_ + *t_N_*)/2 to (*t_N_* + *t_N_*_+1_)/2.

The algorithm normalizes the pulses extracted. On the X-axis (time), each PPG and ECG pulse was normalized to a value between 0 and 400 using the interp1 function in Matlab, while the MW pulse was normalized to a value between 0 and 1250. On the Y-axis, the amplitudes were normalized from 0 to 1 as follows: (*y* − *y_min_*)/(*y_max_* − *y_min_*). The use of normalization is crucial in the analysis of pulse shapes, particularly when comparing them or extracting features. This makes it possible to obtain reliable and comparable results, uncontaminated by external factors or differences between individuals [20,31].

In the context of biomedical signal analysis, it is crucial not only to acquire physiological signals but also to assess their quality. For clinical applications, reliable pulse detection and feature extraction are essential [33]. Therefore, a signal quality control procedure was implemented prior to feature extraction. For PPG and MW signals, pulse quality was assessed using a Signal Quality Index (SQI) based on eight quality metrics described in [34]. Only pulses with SQI ≥ 0.9 were retained for further analysis, while lower-quality pulses were excluded from these analyses. Their rejection was reflected in the reported proportion of high-quality pulses for each sensor in Section 3.3. Similarly, ECG pulse quality was evaluated using correlation analysis with a reference pulse. Only ECG pulses with a Pearson Correlation Coefficient (PCC) ≥ 0.8 were considered acceptable and included in subsequent analysis. Although dedicated motion artifact removal algorithms were not applied due to the controlled measurement conditions, the implemented quality assessment procedure enabled algorithmic identification and rejection of low-quality pulses prior to pulse averaging.

The synchronization procedure was based on a common acquisition time reference shared by all recorded signals (ECG, PPG, and MW), ensuring time-aligned data acquisition across modalities. Synchronization was performed at the hardware level, as described in Section 2.5, through a common timing architecture that enabled simultaneous acquisition of all signal channels. All signals were recorded using a unified sampling clock and subsequently resampled to a common time base to facilitate multimodal signal analysis. In the subsequent analysis stage, ECG R-peaks were used as fiducial markers for precise temporal alignment of PPG and MW signals during feature extraction.

### 2.8. Protocol and Characteristics of the Study Group

As part of the experimental trials, MS was tested in 32 volunteers. The tests were conducted in a quiet room at room temperature with neutral lighting in the morning hours, after a minimum of 6 h of rest at night. Twelve hours before the tests, volunteers were advised to refrain from consuming alcohol, coffee, tea, and other substances that significantly affect the autonomic nervous system; volunteers were also advised to refrain from smoking. After giving their written consent to participate in and complete the interview, each volunteer sat on a specially prepared couch, after which the sensors were attached to the volunteer. Once all sensors were in place, a calibration phase was followed to optimally adjust their positioning and ensure the best possible signal quality. This was followed by the main measurement phase, which lasted approximately 1 h. During this phase, the system recorded biomedical signals synchronously and continuously. Once the main phase started, conversations and movement of the operating staff were limited to the absolute minimum. After approximately 1 h, the recording was stopped. The staff then removed the previously attached components of the measurement system and prepared it for the next volunteer. During this time, data was also downloaded from the SD card.

A total of 32 volunteers (14 women and 18 men) aged 21 to 64 years participated in the trials. A detailed description of the study group is presented in Table 1.

No participants reported known cardiovascular or respiratory diseases at the time of data collection. The inclusion criteria for the study were age between 18 and 65 years for both sexes, absence of advanced cardiovascular diseases, and provision of written informed consent to participate in the study. Exclusion criteria included, among others, chronic kidney disease and severe obesity (BMI > 40 kg/m^2^), as well as any condition that could significantly affect the study or prevent stable measurement conditions. All participants provided written informed consent prior to participation. The study protocol was approved by the local bioethics committee.

## 3. Results

### 3.1. Photos of the Developed System

Below are photos of the individual modules of the developed MS. Figure 9 shows the main hub (PSH module) with sensors connected to the front panel. The upper surface of the PSH housing features LEDs that indicate the status of the device, as well as buttons that enable the device to be reset and the measurement to start/stop. The dimensions of the PSH module are 120 mm × 100 mm × 35 mm (width × length × height).

Figure 10 shows an example of a PPG sensor in a housing and an MWPPG sensor, while Figure 11 shows an example of an MW sensor in a shielding housing. The dimensions of the PPG sensors are 60 mm × 55 mm × 18 mm and the MW sensors are 75 × 35 × 12 (width × length × height).

### 3.2. Example Signals Measured During the Trials

Figure 12 shows an example of a segment of recorded PPG and an ECG signals. One PPG channel (from the infrared (IR) LED) is shown for each PPG sensor, and Figure 13 shows segments of signals from the MW sensors.

Figure 14 shows an example of a PPG signal segment from the MWPPG (S3) sensor that uses six LEDs with different light wavelengths ranging from blue to infrared. The visible waveforms differ slightly in shape, which results from the varying depths of penetration of light and the differing absorption by tissues and blood.

Figure 15 shows an example segment of recorded ECG, PPG (IR from S2) and MW (S8) signals, illustrating the synchronization of the MS. The red line represents the first derivative of the PPG, indicating its rate of change. First, the peak of the ECG signal (R wave) is observed, followed by the onset of the PPG signal, and, almost simultaneously, the peak of the MW signal. Physiologically, the ECG signal reflects the electrical excitation of the heart and the ejection of blood into the blood vessels, while the subsequent changes in the PPG and MW signals correspond to the appearance of the pulse wave on the fingers of the left hand—specifically on the index finger (PPG) and the thumb (MW).

### 3.3. Results of Pulse Averaging

After conducting experimental trials in 32 volunteers to compare individual sensors and the impact of their placement on signal shape and quality, the extracted pulses were averaged for the entire study group. Only pulses of very high quality were used for the averaging process (for PPG and MW, SQI ≥ 0.9; for ECG, PCC ≥ 0.8). Figure 16 shows the averaged ECG and PPG pulses from sensors S1–S4 the IR LED.

Additionally, the averaged pulses from individual LEDs of the MWPPG sensor are shown in Figure 17. In this case, no significant differences in pulse shape were observed, and the changes between them are minimal.

Figure 18, on the other hand, shows the averaged pulses from individual MW sensors. In the analysis of MW signals, significant differences in the shapes of the averaged pulses were observed between individuals, as shown in Figure 19.

An analysis of the number of correct and incorrect pulses for individual sensors was also conducted following signal quality assessment (Table 2). The highest percentage of very good quality PPG pulses (SQI ≥ 0.9) was recorded by the S3 sensor (64%), with sensor S2 achieving a slightly lower result (59%). The weakest results were recorded for sensor S1 (20%), while sensor S4 achieved an intermediate result of 30%. Among the MW sensors, sensor S6 achieved the best result (30%), and sensor S7 the lowest (21%). Between them were sensors S5 (27%) and S8 (28%). For the ECG signal, in a group of 32 volunteers, the percentage of very high-quality beats was as high as 75%.

Additional analysis of signal quality for different sensors and measurement locations is presented in Table 3. For each sensor, parameters describing signal morphological quality and pulse detection performance were calculated, including the signal-to-noise ratio (SNR), relative spectral quality index (RSQI), entropy, nonstationarity parameter (NS), amplitude distribution skewness (Skewness), and pulse detection metrics with respect to the reference ECG signal (Precision, Recall, and F1-score). The SNR was calculated from the raw signals using the conventional power-based definition. The power spectral density was estimated using Welch’s method. The signal power (P_signal_) was defined as the spectral power within the cardiac frequency band (0.7–3.0 Hz), while the noise power (P_noise_) was calculated as the remaining spectral power within the analyzed frequency range (0.1–20.0 Hz). The SNR was then computed as 10log_10_(P_signal_/P_noise_). Results are reported as mean (ME) ± standard deviation (SD). The 95% confidence intervals for the mean values, shown in parentheses, were calculated using Student’s t-distribution based on the standard error of the mean (SEM = SD/√*n*), where *n* is the number of observations. For statistical comparisons, pulse-level measurements were first aggregated at the participant level for each sensor, and the resulting subject-level values were used in the Friedman test. Therefore, the effective sample size corresponded to the 32 participants included in the analysis. A significance level of *p* < 0.05 was assumed. The Friedman test was used to assess overall differences between sensor locations and did not identify specific pairwise differences between individual sensors.

The highest PPG signal quality was obtained for sensors placed on the fingers and forehead. The highest SNR value was achieved by sensor S4 located on the forehead (12.61 ± 3.98 dB). Sensors S2 and S3, positioned on the index and middle fingers, respectively, also demonstrated high signal quality, achieving SNR values of 12.28 ± 2.19 dB and 12.19 ± 2.45 dB. All PPG sensors exhibited very high RSQI values (~ 0.99), confirming a strong dominance of the cardiac-related component. In addition, sensors S2 and S3 achieved the highest Recall (0.67 ± 0.24 and 0.67 ± 0.21, respectively) and F1-score (0.74 ± 0.21 and 0.74 ± 0.17).

For MW sensors, substantially lower parameter values were observed. SNR for MW sensors ranged from 5.91 ± 0.98 dB (S5) to 7.95 ± 1.26 dB (S7). Although signal morphology quality was lower, RSQI values remained relatively high (0.91–0.94), indicating that cardiac rhythm information was still present in the recorded signals. The best MW performance was achieved for locations above the brachial artery (S6) and radial artery (S7), which exhibited the highest RSQI values (0.93–0.94), as well as the highest Recall (0.27 ± 0.14 and 0.29 ± 0.14) and F1-score (0.41 ± 0.16 and 0.43 ± 0.15). The poorest performance was observed for sensor S5 placed on the chest above the heart, which exhibited the lowest SNR (5.91 ± 0.98 dB), lowest Recall (0.21 ± 0.06), and lowest F1-score (0.33 ± 0.07).

### 3.4. Initial PAT Analysis

One of the important parameters used in cardiovascular monitoring is PAT. It can be used to estimate BP and pulse wave velocity measure, and its values can also confirm the synchronization of multimodal measurements. Figure 20 shows the ECG, PPG and MW signals with marked fiducial points used to determine the PAT. In our case, PAT is defined as the time between the maximum of the R wave of the ECG signal and the local minimum of the PPG or MW signal. For the MW signal, the local minimum was selected as a consistently identifiable fiducial point corresponding to the onset region of the pulse waveform; this is analogous to the pulse-foot definition commonly used in PPG analysis.

Figure 21 presents the average PAT values obtained for all 32 volunteers using the two PPG sensors (S3 and S4) and the MW sensor (S6).

The measured delays confirm the temporal synchronization between the ECG and PPG/MW pulse, indicating that the proposed system is suitable for future PAT-related investigations.

## 4. Discussion

### 4.1. Analysis of Sensors in the Developed Measurement System

The shape of the pulses varies at each level of the arterial tree, depending on regional differences in arterial anatomy or blood flow [35]. Therefore, the PPG and MW sensors of the developed measurement system were placed at multiple body locations to evaluate their signal quality and suitability for further cardiovascular analysis. The Friedman test indicated overall differences between sensor locations for signal quality and pulse detection performance; however, no pairwise statistical comparisons between individual sensors were performed. Among the PPG sensors, the best results were obtained at locations characterized by high tissue perfusion and stable sensor-to-skin contact, namely the fingers and forehead. This observation is consistent with cardiovascular physiology, as the fingertips are highly vascularized and allow transmissive measurements through the entire tissue volume, while the forehead provides excellent perfusion and is less susceptible to contact pressure variations than the wrist. Sensors S2 and S3, placed on the index and middle fingers, respectively, achieved high SNR values (12.28 ± 2.19 dB and 12.19 ± 2.45 dB), very low heart rate estimation errors relative to the reference ECG signal (0.85 ± 0.53 BPm and 0.82 ± 0.58 BPm), and the highest Recall (0.67 ± 0.24 and 0.67 ± 0.21) and F1-score (0.74 ± 0.21 and 0.74 ± 0.17). These results indicate that finger-based measurements provide not only high morphological signal quality but also the most reliable pulse detection performance. An additional advantage of finger measurements was the frequent presence of a clearly visible dicrotic notch, which provides valuable diagnostic information regarding aortic valve closure and arterial elasticity. The highest morphological quality parameters were obtained for sensor S4 located on the forehead. This sensor achieved the highest SNR (12.61 ± 3.98 dB), the highest entropy (5.75 ± 0.08), and one of the lowest nonstationarity values (NS = 0.56 ± 0.06). These findings indicate excellent signal quality, high pulse morphology repeatability, and strong temporal stability. The result is consistent with previous reports demonstrating that forehead PPG signals are typically highly stable due to the rich vascularization of this region. Sensor S1 placed on the wrist achieved lower SNR values (8.66 ± 2.17 dB) and the highest HR estimation error among all PPG sensors (3.40 ± 3.32 BPm). These findings are consistent with the well-known susceptibility of wrist-based measurements to motion artifacts, contact pressure variations, and lower peripheral perfusion. Nevertheless, signal quality remained sufficient for reliable analysis, as reflected by the high Precision and F1-score values.

Among the MW sensors, the best results were obtained for locations directly above major arteries. Sensor S6 positioned over the brachial artery and sensor S7 positioned over the radial artery achieved the highest Recall (0.27 ± 0.14 and 0.29 ± 0.14) and F1-score (0.41 ± 0.16 and 0.43 ± 0.15) values among all MW sensors. Sensor S7 also achieved the highest RSQI (~0.94), while S6 exhibited one of the highest SNR values within the MW group. These findings confirm that locations directly above major arteries facilitate more effective detection of blood volume changes associated with pulsatile blood flow than other measurement sites. This indicates reduced pulse morphology repeatability and increased susceptibility of microwave signals to noise and physiological artifacts. Although Precision remained relatively high (~0.90), indicating that most detected pulses were correct, a considerable proportion of true cardiac events remained undetected. The weakest performance was observed for sensor S5 positioned on the chest above the heart. This location exhibited the lowest SNR 5.91 ± 0.98 dB), lowest Recall (0.21 ± 0.06), and lowest F1-score (0.33 ± 0.07). Despite its proximity to the source of cardiac activity, this location is particularly susceptible to respiratory motion and chest wall displacement, which significantly complicate stable microwave signal acquisition.

The descriptive results showed generally higher signal-quality metrics for PPG sensors than for MW sensors. PPG sensors exhibited higher entropy values (5.61–5.75), indicating richer signal structure, as well as higher NS values (0.56–0.65), which, together with high SNR, can be interpreted as preserving natural physiological variability while maintaining signal quality. In contrast, MW sensors exhibited lower entropy, lower NS values, and greater variability in amplitude distribution skewness. Combined with negative SNR values and lower pulse detection performance, these findings suggest reduced morphological stability and greater susceptibility of microwave signals to external disturbances. Overall, the results indicate that the most favorable locations for PPG measurements are the index finger, middle finger, and forehead, whereas the most promising locations for microwave measurements are above the brachial and radial arteries.

### 4.2. Analysis of Averaged Pulses

The presented averaged ECG pulses is of very high quality—the individual QRS complex waves and the T wave are clearly recognizable. Analysis of PPG signals indicates that the flattest waveform occurs with the sensor placed on the wrist (S1). This is associated with weaker peripheral perfusion in this area, which often results in the absence of a clearly defined dicrotic notch in the signal. The situation is different for the other sensors, where the dicrotic notch is already visible. The shape of the PPG pulses from the sensors S2 and S3 is very similar, resulting from their similar measurement locations (index and middle fingers). In this case, the sensor type—transmissive or reflective—has no significant impact on the shape of the recorded signal. However, the PPG pulse from sensor S4 takes on a shape intermediate between the signals recorded on the fingers and the signal from the wrist. A slight dicrotic notch can also be observed in its waveform, though its character differs from the signals from the fingers.

In the case of averaged MW pulses, it can be observed that their shapes are similar to each other, yet they clearly differ from PPG pulses. The MW waveform should be interpreted as a cardiac-synchronous signal rather than as a direct measure of blood flow dynamics, since the present study does not allow the contributions of blood volume changes, tissue motion, respiration, and other mechanical effects to be distinguished. Moreover, the observed similarity between the MW waveform and the first derivative of the PPG signal may suggest that the MW sensor is sensitive to dynamic tissue changes associated with the cardiac cycle. This similarity may also be physically plausible considering the Doppler-based operating principle of the MW sensor, which makes it sensitive to motion and velocity-related changes within the investigated tissue. However, this interpretation remains a hypothesis, as waveform similarity alone does not establish the underlying physiological mechanism and no independent physiological reference was used in the present study. MW signals also allow for accurate detection of pulse onset, although their waveform has a significantly different character. It should be noted that in the analysis of the MW signals, significant differences in the shapes of the averaged pulses were observed between individuals, as shown in Figure 19. In many cases, the pulse exhibits a clearly defined peak with steep rising and falling edges, while in others it appears considerably more flattened (as in Figure 18). This may result, among other things, from anatomical differences in tissues (varying thickness and structure affect the way the wave propagates and reflects), hemodynamic properties (e.g., arterial stiffness, pressure), sensor positioning relative to the vessel, and the dynamics of the pulse wave upstroke, whose velocity is being measured.

### 4.3. Comparison of PPG and MW Technologies

The PPG and MW technologies utilize different physical phenomena for measurement. PPG is based on the recording of changes in the intensity of light emitted into the tissue and absorbed or reflected by the blood circulating in the vessels. In contrast, MW technology is based on the Doppler effect. An MW signal emitted toward the tissue interacts with flowing blood, reflects, and returns to the sensor. The reflected signal is characterized by a change in frequency and phase relative to the transmitted signal. A significant advantage of MW technology over PPG is its lower susceptibility to external conditions, such as ambient lighting, as well as the lack of influence from skin color or the presence of intervening layers, which allows measurements to be taken even through thin clothing. At the same time, the MW signal can provide information from deeper tissue structures, making it potentially complementary to PPG methods. Moreover, by appropriately selecting the microwave frequency, the penetration depth of the electromagnetic wave can be adjusted. In contrast, the probing depth in PPG is largely constrained by the availability of LEDs operating at specific wavelengths, which limits the flexibility of tailoring the measurement depth to a particular application.

However, the limitations of MW technology in the context of wearable devices include relatively higher energy consumption (in the case of the developed sensors, approximately 10 times higher than PPG), the need for precise sensor placement relative to arterial vessels (placement dependency), greater sensitivity to motion artifacts, including muscle activity and body movements, and larger dimensions compared to PPG sensors. Additionally, MW measurements exhibit inter-subject variability due to differences in tissue composition and anatomical structure, which may affect signal consistency between individuals. Although MW technology is currently still in its early stages of development, the use of modern sensors indicates real potential for further miniaturization, reduced power consumption, and the application of this technique.

Although the MW sensors showed a lower proportion of high-quality pulses than the best-performing PPG sensors, MW sensing is based on a different physical mechanism and may therefore provide information related to dynamic changes in tissue dielectric properties and motion. In addition, unlike optical PPG measurements, MW sensing is not directly affected by ambient light conditions, which may be advantageous in selected measurement scenarios. Despite these advantages, MW sensors do not currently constitute a direct alternative to PPG techniques, but rather a complement, requiring further research on signal stability, circuit miniaturization, and effective integration with other measurement modalities. Moreover, the present study does not demonstrate that the inclusion of MW sensing improves the performance of PPG/ECG-based monitoring, and this potential incremental benefit requires further investigation.

### 4.4. Analysis of the Application of Multi-Site Measurement

The use of a multi-site measurement architecture may offer a potential advantage in terms of the robustness of estimated physiological parameters against local artifacts and signal degradation. In single-point systems, their quality is highly dependent on measurement conditions at a given location, making them susceptible to interference related to movement, changes in perfusion, or inaccurate sensor placement. In the proposed solution, parallel signal acquisition from multiple body sites may facilitate the identification and compensation of local disturbances, as well as the selective use of the most reliable information sources at any given moment. This approach may contribute to a more stable extraction of temporal and morphological features that could potentially be used to estimate various parameters, including HR, SpO_2_ and BP. However, the robustness of the multi-site architecture against local artifacts and its ability to compensate for degraded signals were not directly evaluated in the present study.

### 4.5. Multispectral PPG Measurement

The use of multispectral PPG (MWPPG) enables the simultaneous recording of optical signals at several wavelengths, allowing for the observation of changes in blood volume at various tissue depths and in different layers of the vascular system. This approach increases the amount of available physiological information and can improve resistance to changes in local perfusion, skin temperature, or individual optical properties of tissues. At the same time, MWPPG involves greater hardware and algorithmic complexity as well as higher power consumption.

### 4.6. Multimodal Measurement

Recently, there has been dynamic development in the application of multimodal approaches in medical diagnostics. Such approaches enable the simultaneous acquisition of information from different sensing modalities, reflecting complementary aspects of cardiovascular system function. The PPG signal provides information on changes in blood volume in the peripheral microcirculation, enabling pulse wave morphology analysis and feature extraction. The shape, amplitude, and temporal relationships of fiducial points in the PPG waveform reflect vascular properties and pulse wave propagation dynamics, which may support pulse wave analysis.

In turn, the ECG signal provides information about the electrical activity of the heart and the timing of ventricular contraction initiation. Changes in QRS complex morphology, time intervals (e.g., QT and RR), and HRV parameters reflect autonomic nervous system activity and cardiovascular load. Simultaneous acquisition of ECG and PPG signals enables determination of temporal cardiovascular parameters such as Pulse Arrival Time (PAT) and Pulse Transit Time (PTT), which are associated with arterial stiffness and cardiovascular risk assessment.

Supplementing these data with signals acquired from microwave (MW) sensors enables observation of deeper tissue and provides a contactless modality for cardiopulmonary monitoring. This modality could potentially improve monitoring robustness in situations where optical measurements are degraded; however, this potential benefit was not evaluated in the present study and requires dedicated experimental validation. From a clinical perspective, MW sensing can support continuous monitoring of heart and respiratory activity without direct skin contact, which is particularly beneficial in long-term patient observation, in patients with limited mobility, or in scenarios where conventional wearable sensors are not feasible. This additional modality may therefore enhance the applicability of the system in remote and ambulatory healthcare settings.

The integration of ECG, multispectral and multi-site PPG, and MW sensing may increase the diversity of cardiovascular-related information captured from different sensing modalities, providing a richer and more comprehensive dataset for clinical and research applications. Whether such multimodal integration improves the stability, robustness, or diagnostic reliability of cardiovascular assessment remains to be demonstrated in future studies.

### 4.7. Review of Other Similar Works

Table 4 provides a summary of other similar works on multimodal and multi-site measurement systems used for biomedical signal measurement. The presented literature demonstrates a wide variety of measurement systems used to measure biomedical signals for vital sign monitoring, both in terms of the signals recorded and the sensor locations. Most of the works analyzed are based on PPG and ECG signals, supplemented, among others, by Impedance PPG (IPG), Seismocardiography (SCG) or skin temperature (ST). Single-point solutions or those with limited sensor distribution predominate. The number of study groups conducting their own trials is generally small (approx. 10–15), and the duration of measurement sessions is relatively short (approx. 10–15 min), indicating that many of the presented systems remain in the prototype research stage. Based on this overview, a trend toward multimodal measurements is evident; however, the use of different modalities with a multi-site architecture and full experimental validation remains rare, underscoring the need for further research in this area. Furthermore, the use of MW sensors for biomedical signals recording remains limited compared to commonly used techniques, such as optical methods. The number of works that use this type of sensor in the context of monitoring physiological parameters is very small—only one work addresses the use of a millimeter-wave radio frequency sensor. This indicates that the potential of this technology is not yet adequately recognized and highlights a research gap that justifies further research on the use of MW sensors in health monitoring systems.

## 5. Capabilities of the Proposed Measurement System

### 5.1. Long-Term Monitoring of Biomedical Signals

Due to its non-invasive nature and user comfort, the proposed system can be used for long-term (multi-hour) monitoring of biomedical signals in controlled or semi-naturalistic conditions. The multi-site placement of sensors allows for flexible adaptation of the configuration to the measurement scenario and user preferences, which enhances the comfort and acceptability of the system. With further miniaturization and optimization of sensors, the system may be extended to ambulatory use in more natural daily-life conditions. This approach is particularly important in the context of trend monitoring, detecting episodes such as hypertension, and assessing the response of the cardiovascular system to daily activities.

### 5.2. Data Fusion for Medical Diagnostics

The proposed MS architecture also provides a solid foundation for the further development of multimodality data fusion methods. Access to synchronized, multimodal, and multi-site signals enables the implementation of advanced feature selection strategies, adaptive weighting of information sources, and models that account for individual hemodynamic differences. The dataset collected in this way offers extensive opportunities for research using ML/DL methods aimed at identifying complex relationships between biomedical signals and the physiological parameters of the body. In particular, multimodal data analysis can enable the search for new diagnostic indicators related not only to cardiovascular function but also to other physiological disorders, such as metabolic diseases or changes associated with body aging processes. Integrating measurement data with demographic and clinical information from trial participants can further enable investigation of the impact of population-level factors on biomedical signal characteristics and the development of various disease entities. In the context of future research, this approach may contribute to the development of more universal diagnostic models that support early disease detection, health monitoring, and a better understanding of the physiological and hemodynamic mechanisms observed in multimodal data.

### 5.3. Non-Invasive Measurement Systems for Continuous Medical Diagnostics

Currently used diagnostic devices, despite their widespread use in clinical practice, often have significant limitations when it comes to long-term and continuous monitoring of physiological parameters. Many measurement methods commonly used in clinical practice require the use of uncomfortable components or periodic measurements, which can be a burden on the user and affect comfort during daily activities or sleep. As a result, long-term trials conducted using such methods may be associated with a reduction in the quality and reliability of the data obtained. Additionally, periodic measurements provide only intermittent information about the state of the body, which limits the ability to observe dynamic changes that occur in the cardiovascular system and other physiological systems. Consequently, short-term episodes of changes in physiological parameters associated, for example, with a stress response, a change in body position, or physical exertion, may go unrecorded.

However, the development of modern measurement techniques enables the continuous and non-invasive recording of biomedical signals while maintaining a high level of user comfort. The continuous acquisition of physiological signals allows not only the estimation of selected diagnostic parameters but also the analysis of their trends and the identification of sudden changes in the condition of the body.

In this context, MS like this may directly contribute to wearable healthcare systems by enabling multi-sensor integration within compact monitoring platforms capable of continuous operation in daily-life conditions. Such systems also support remote patient monitoring by allowing long-term acquisition of physiological data outside clinical environments, enabling continuous supervision of patients in home and ambulatory settings without the need for frequent clinical visits. In cardiovascular disease screening, such systems facilitate large-scale and repeated measurements, improving the availability of longitudinal data for population-level risk assessment.

Furthermore, the proposed approach may support early diagnosis applications by enabling continuous tracking of subtle physiological changes over time, rather than relying on isolated measurements. This allows earlier identification of deviations from baseline cardiovascular function and supports preventive monitoring strategies aimed at detecting disease progression at an early stage, before the onset of clinically evident symptoms.

It is precisely these capabilities that constitute one of the main motivations for the development of non-invasive measurement systems intended for continuous medical diagnostics and long-term health monitoring in everyday settings—e.g., the solution proposed in this work.

### 5.4. Potential Application of the Measurement System for Cuffless BP Estimation

With the developed MS, it is possible, among other things, to analyze BP using both two-point measurement methods (PAT and PTT) and single-point measurement methods, such as Pulse Wave Analysis (PWA) PPG. The PTT and PAT methods offer a stronger physiological basis because they directly relate to the pulse wave velocity along the vessels, which is linked to arterial stiffness and BP levels; however, they require precise synchronization of signals from at least two locations. Another drawback of pulse wave velocity-based measurement is its sensitivity to changes in other cardiovascular parameters, such as arterial stiffness or Pre-Ejection Period (PEP). In contrast, single-point methods are characterized by simpler implementation and greater potential for integration with wearable devices, but their effectiveness is strongly dependent on the quality and stability of the PPG signal and on the correct identification of pulse morphological features, which may be distorted in the presence of artifacts or changes in peripheral perfusion. In practice, the two-point and single-point approaches should be considered complementary, and combining them within multimodal systems can increase the robustness and reliability of BP estimation under real-world conditions. The advantages of both measurement methods include the ability to measure during daily activities and at night, as well as continuous measurement. When using ECG and PPG signals, other advantages include the low complexity and cost of the measurement system, as well as the minimal computational and memory requirements for processing compared to, for example, MW technology.

### 5.5. Wearable Measurement System

Although the current system is not intended for wearable use due to the number of sensors and measurement locations employed, the proposed architecture provides a valuable framework for analysis of individual sensing modalities to cardiovascular monitoring. After identifying the most informative sensor configurations and reducing system complexity, the development of more compact and wearable multimodal monitoring solutions is enabled. Following further analysis of the acquired sensor data, the proposed system could be optimized and simplified into a wearable form factor while preserving the most clinically relevant information.

## 6. Conclusions and Future Work

This paper presents a multimodal, and multi-site measurement system for synchronous measurement of biomedical signals, integrating PPG sensors, active MW sensors, and ECG signals. The system was evaluated in experimental trials conducted in a group of 32 volunteers. The study demonstrated the feasibility of multimodal, multi-site, and synchronized acquisition of signals from multiple body locations.

Based on the recorded data, pulses from individual sensors were averaged to compare their shape and quality depending on the technology and location of the measurement. The highest-quality signals were recorded from PPG sensors placed on the fingers and from an MW sensor placed on the brachial artery (biceps). The analysis confirmed the possibility of synchronous signal recording from multiple body locations. Based on the analyses, the measurement technologies used were compared in terms of signal quality, location, user comfort, and complexity.

The present evaluation was performed exclusively under stationary conditions. Therefore, the reported results do not characterize the robustness of the proposed method to motion artifacts, nor do they demonstrate clinical utility, reliable estimation of cardiovascular parameters, or an incremental advantage of MW sensing over more established technologies. Evaluating its motion tolerance will be the subject of future work.

The present study has several limitations. The experiments were conducted in a relatively small group of primarily healthy volunteers under controlled stationary conditions, without movements representative of daily-life activities or participants with cardiovascular diseases. In addition, no external clinical reference was used, the MW sensing performance was relatively limited and may depend on sensor positioning, and the derived parameters were not independently validated. Therefore, further studies under conditions more representative of real-world continuous monitoring are required.

Future work may focus on expanding experimental validation, miniaturizing measurement channels, and developing methods for data fusion and personalized medical diagnostics. Furthermore, based on the recorded data, an analysis should be conducted to assess the correlation between the characteristics of individual modalities and the parameters of the cardiovascular system, as well as the feasibility of their accurate and stable estimation. Further studies are also required to determine whether MW sensing provides additional clinically relevant information beyond that obtained from established ECG and PPG measurements.

## Figures and Tables

**Figure 1:**
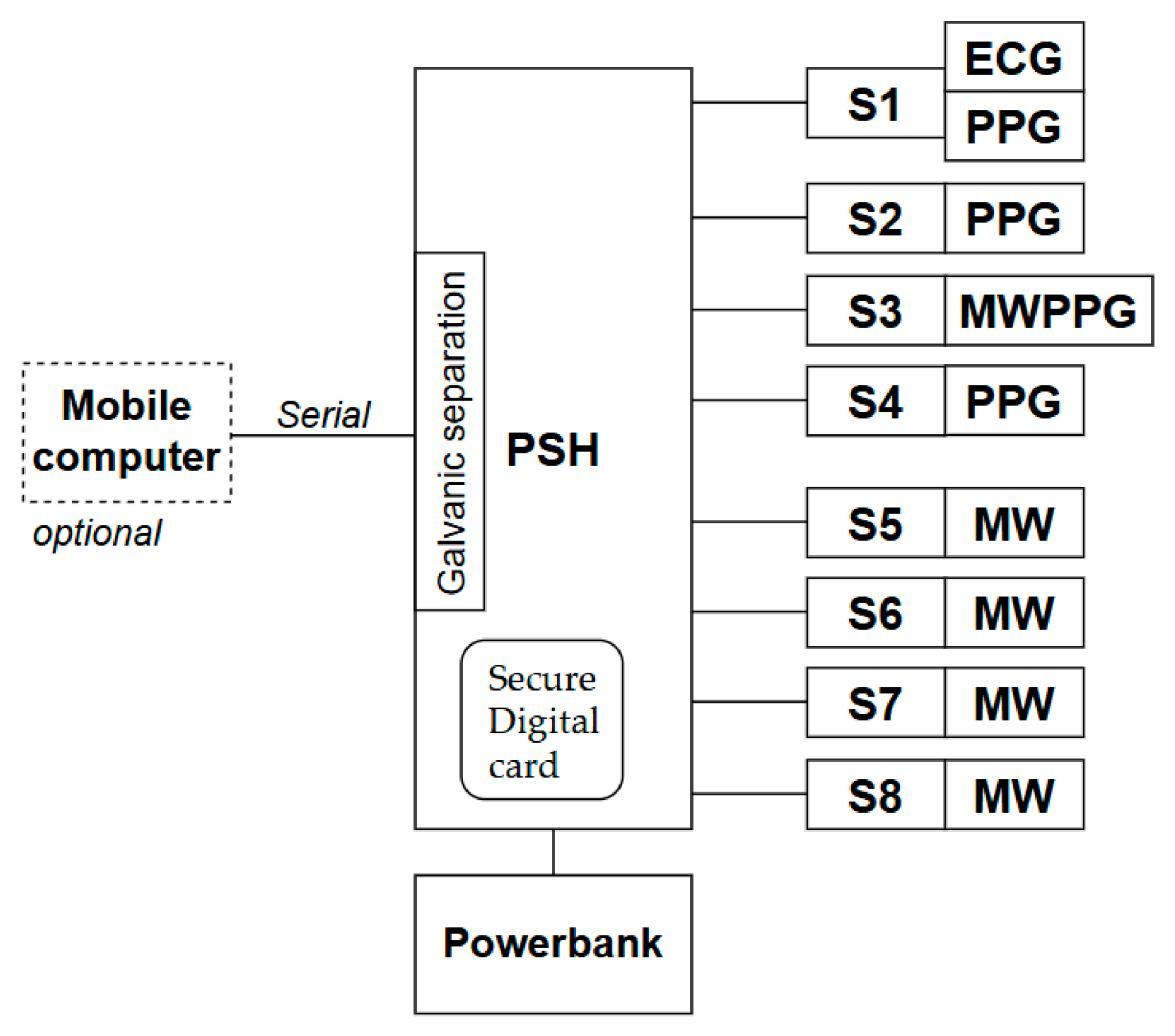
Block diagram of the measurement system.

**Figure 2:**
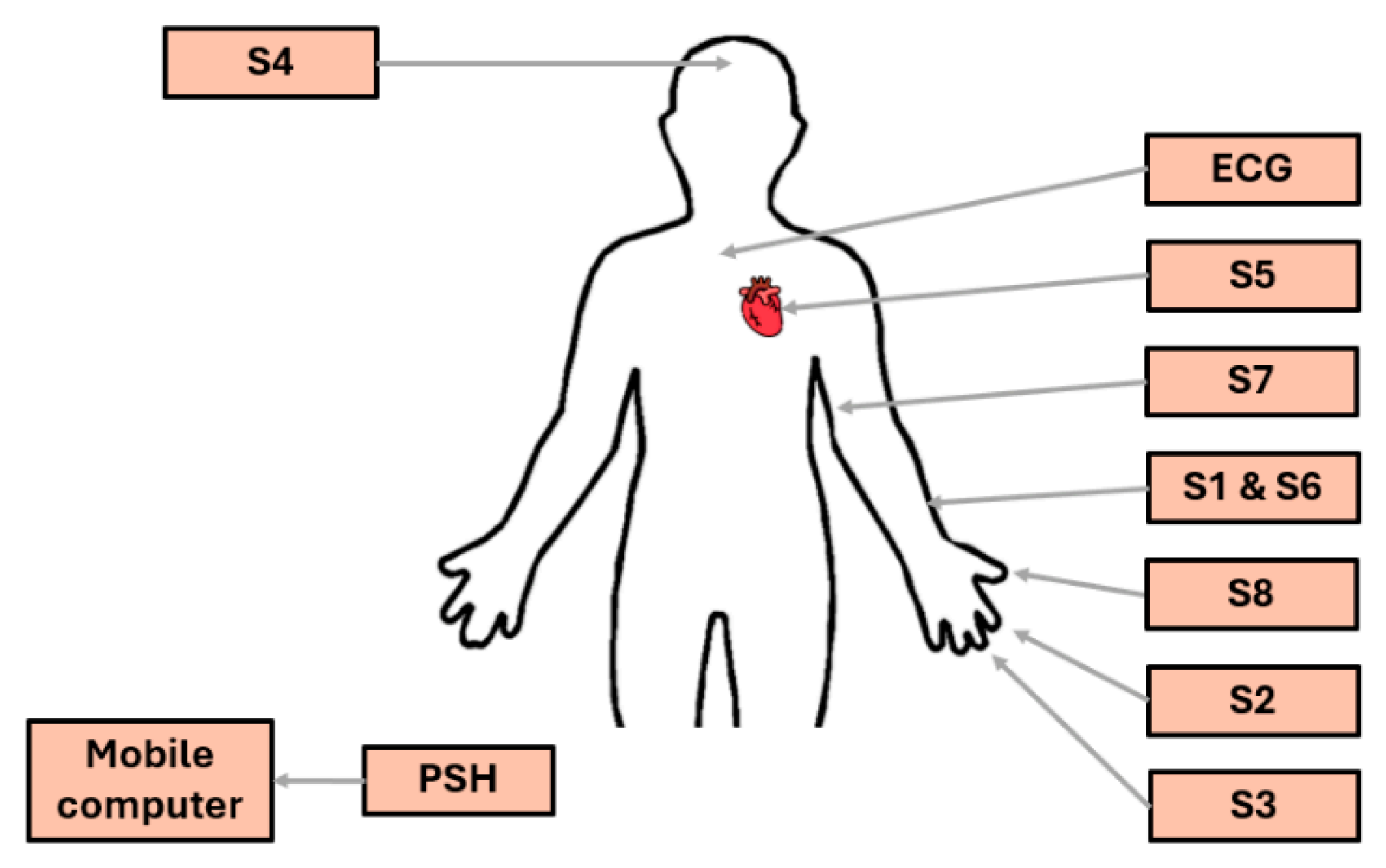
Arrangement of the components of the measurement system.

**Figure 3:**
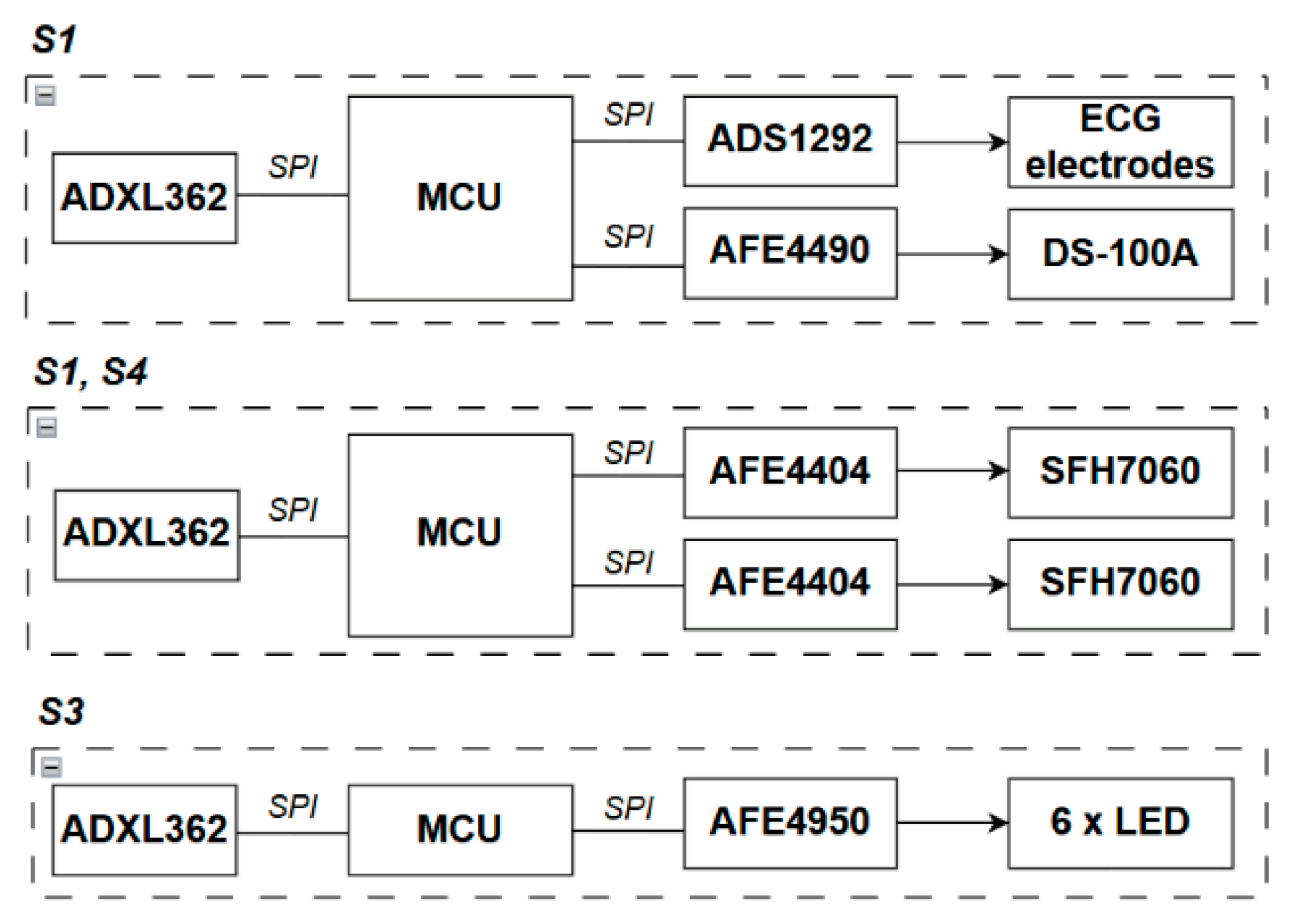
Block diagram of PPG sensors.

**Figure 4:**
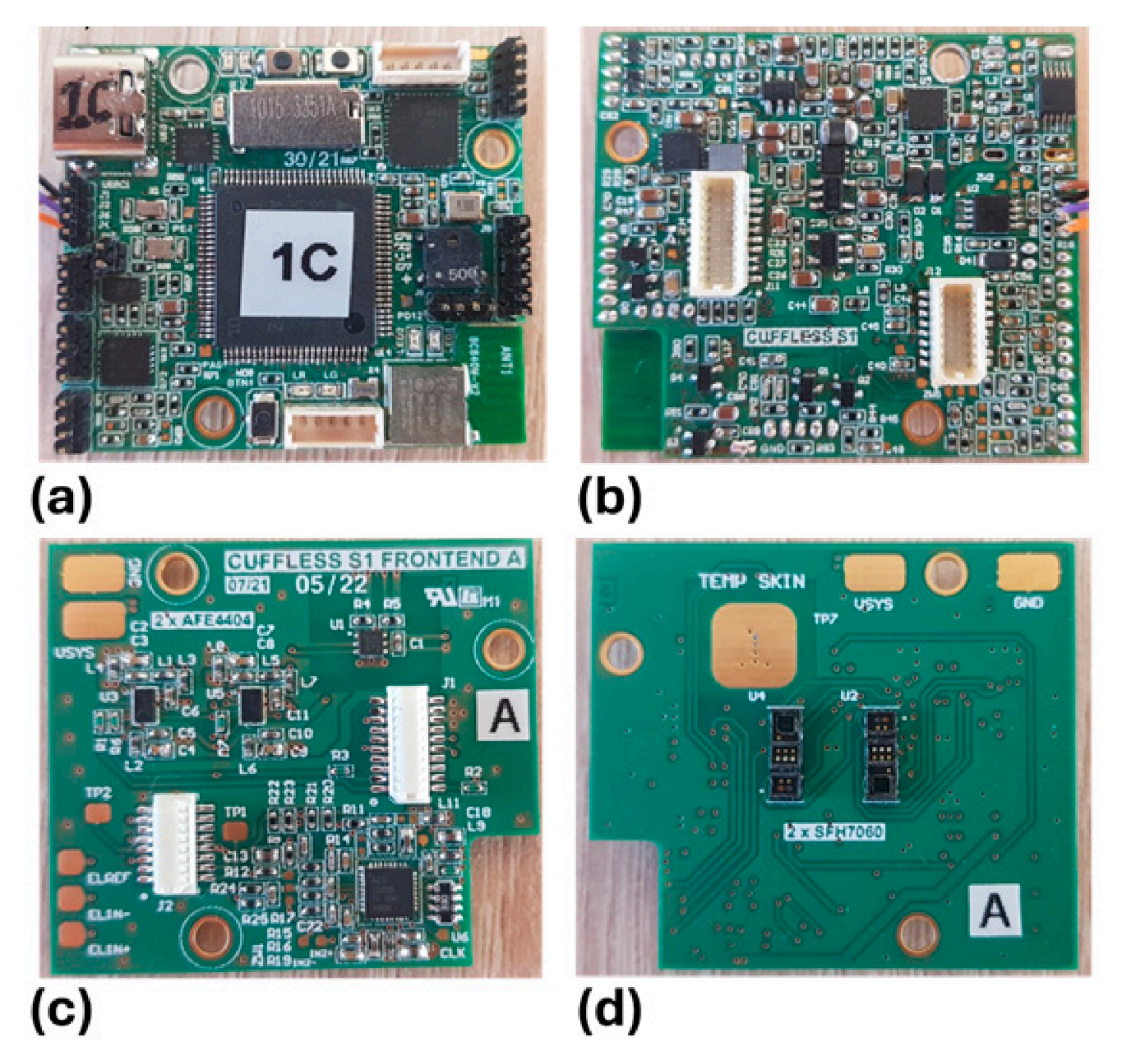
Example PPG sensor: (**a**) top view of the main PCB; (**b**) bottom view of the main PCB; (**c**) top view of the measurement PCB; and (**d**) bottom view of the measurement PCB.

**Figure 5:**
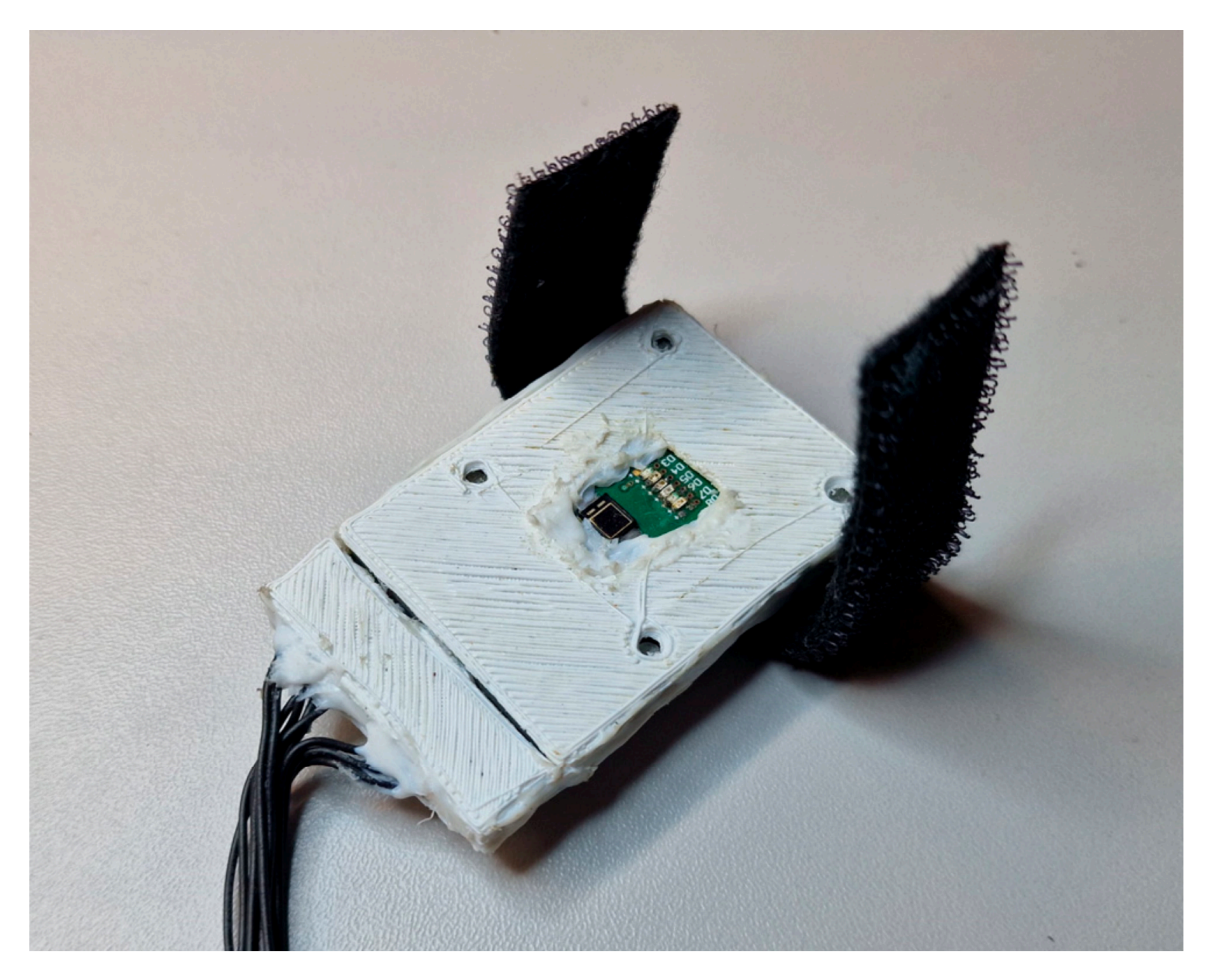
MWPPG sensor view.

**Figure 6:**
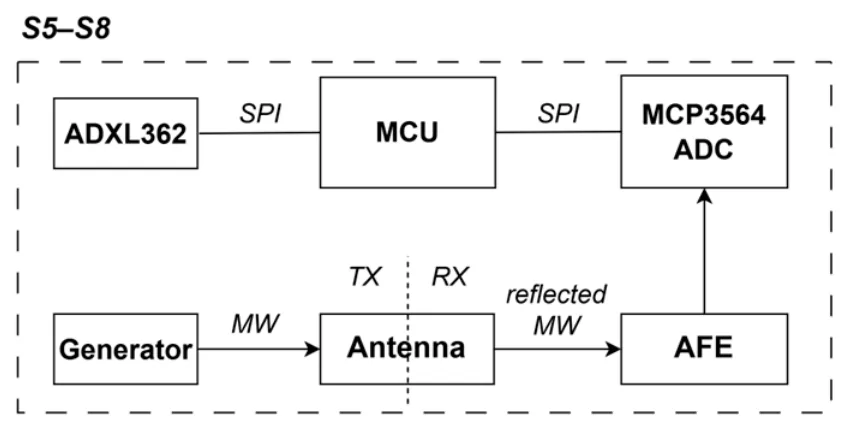
Block diagram of MW sensors.

**Figure 7:**
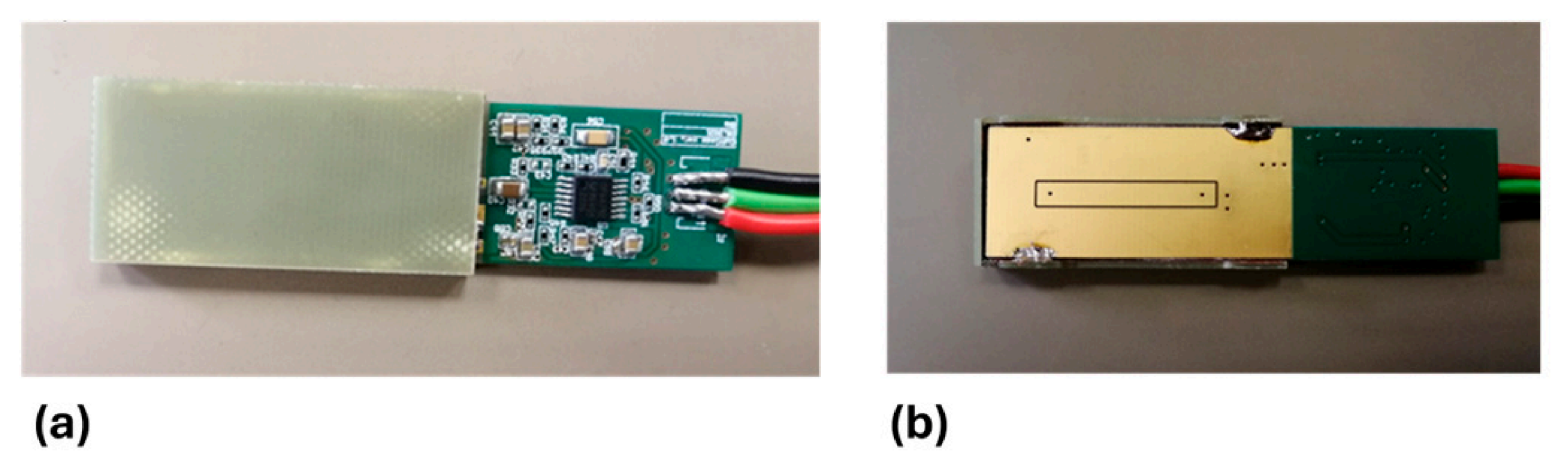
MW probe with integrated antenna and shielding: (**a**) top view; (**b**) bottom view.

**Figure 8:**
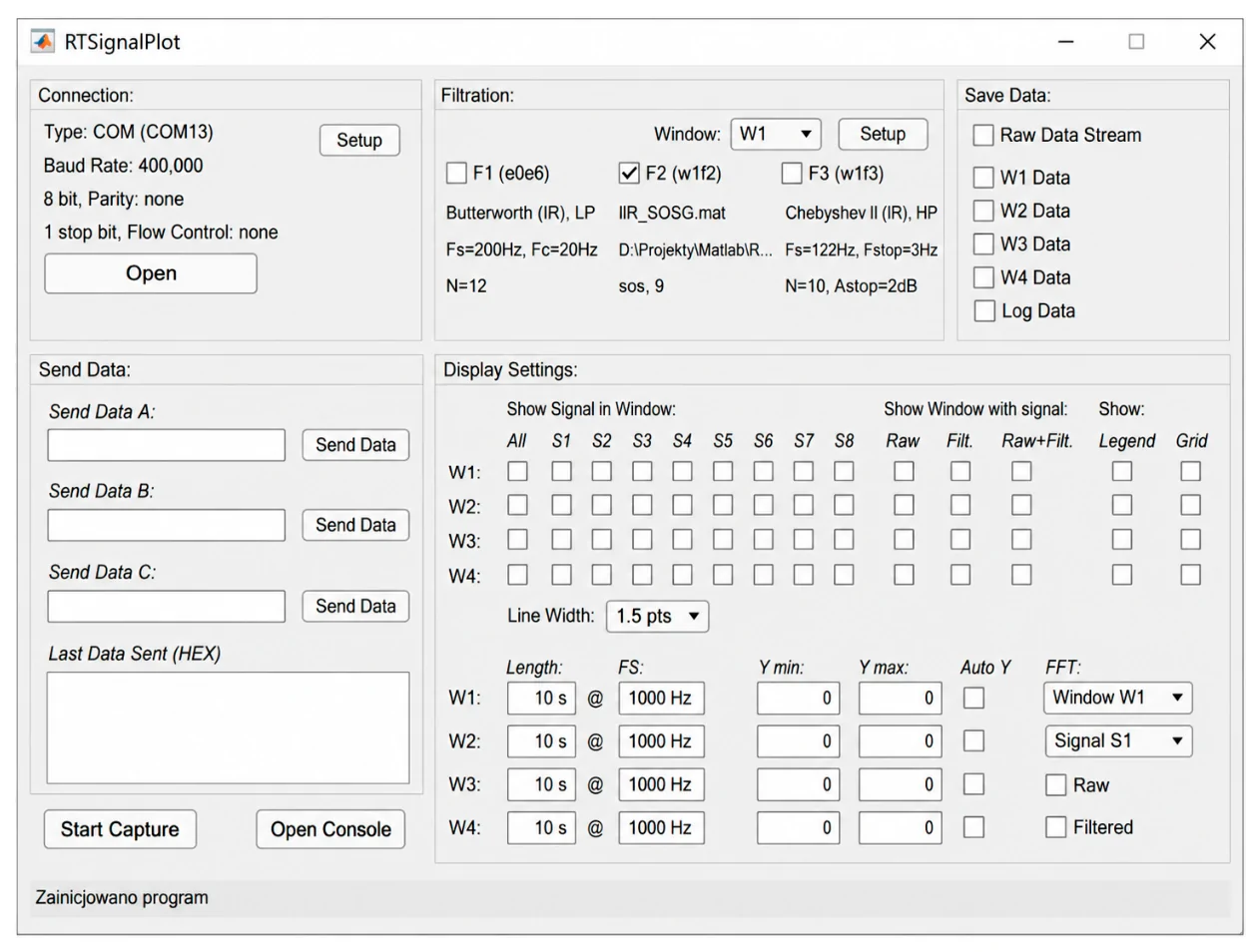
RTSignalPlot application main menu.

**Figure 9:**
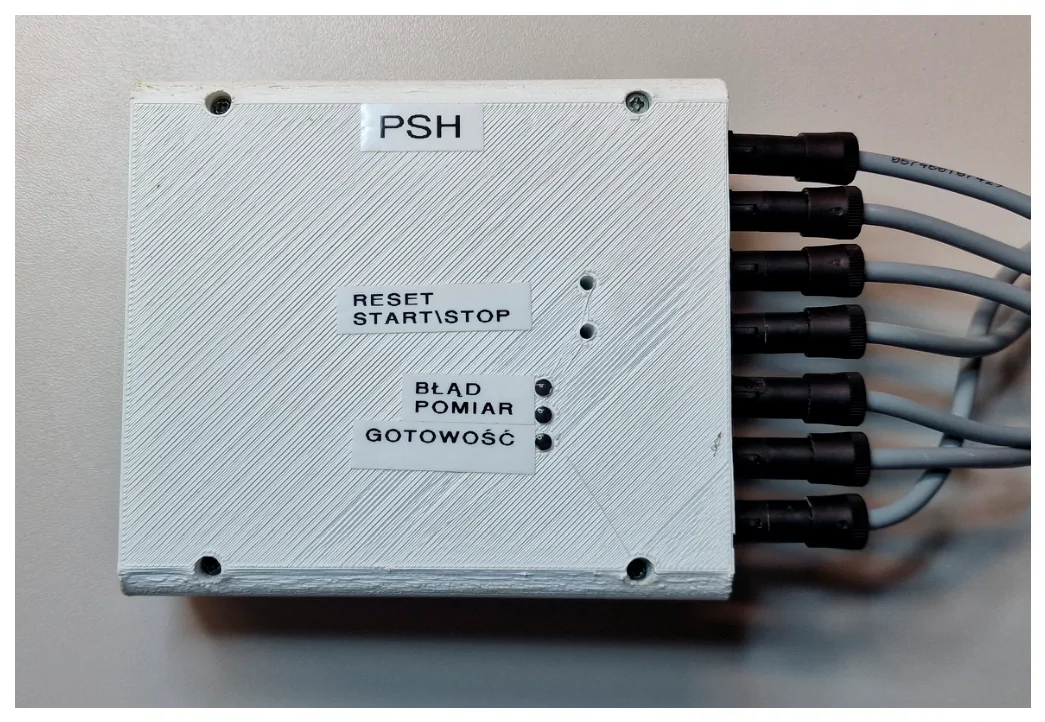
PSH module in a housing.

**Figure 10:**
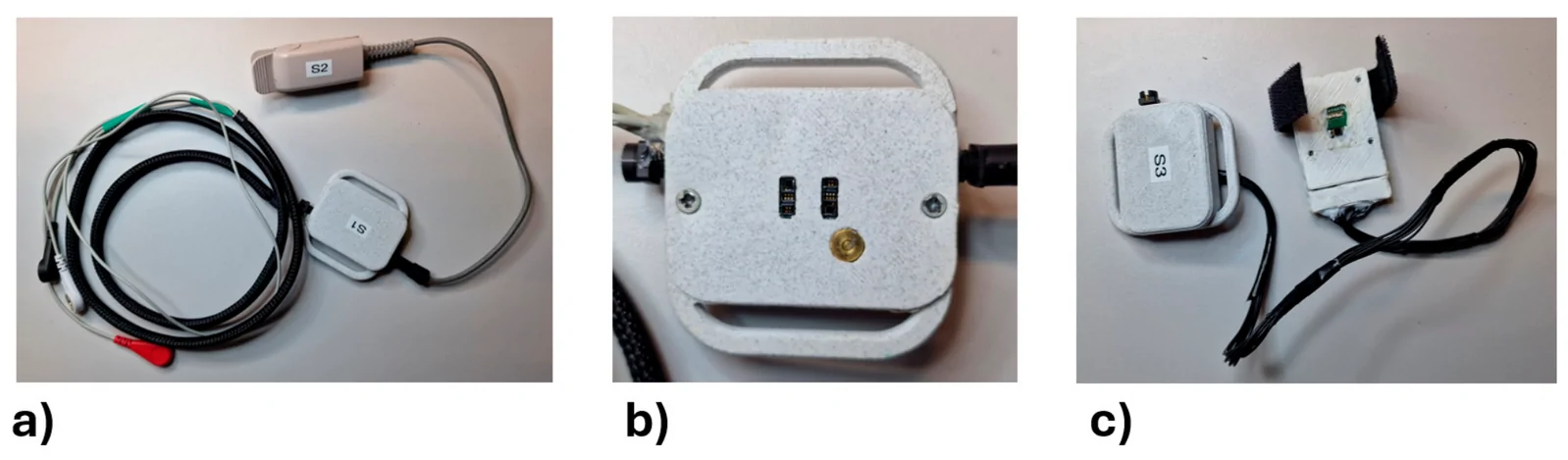
Example of a PPG sensor in a housing: (**a**) S1 & S2 top view; (**b**) bottom view; (**c**) S3 (MWPPG).

**Figure 11:**
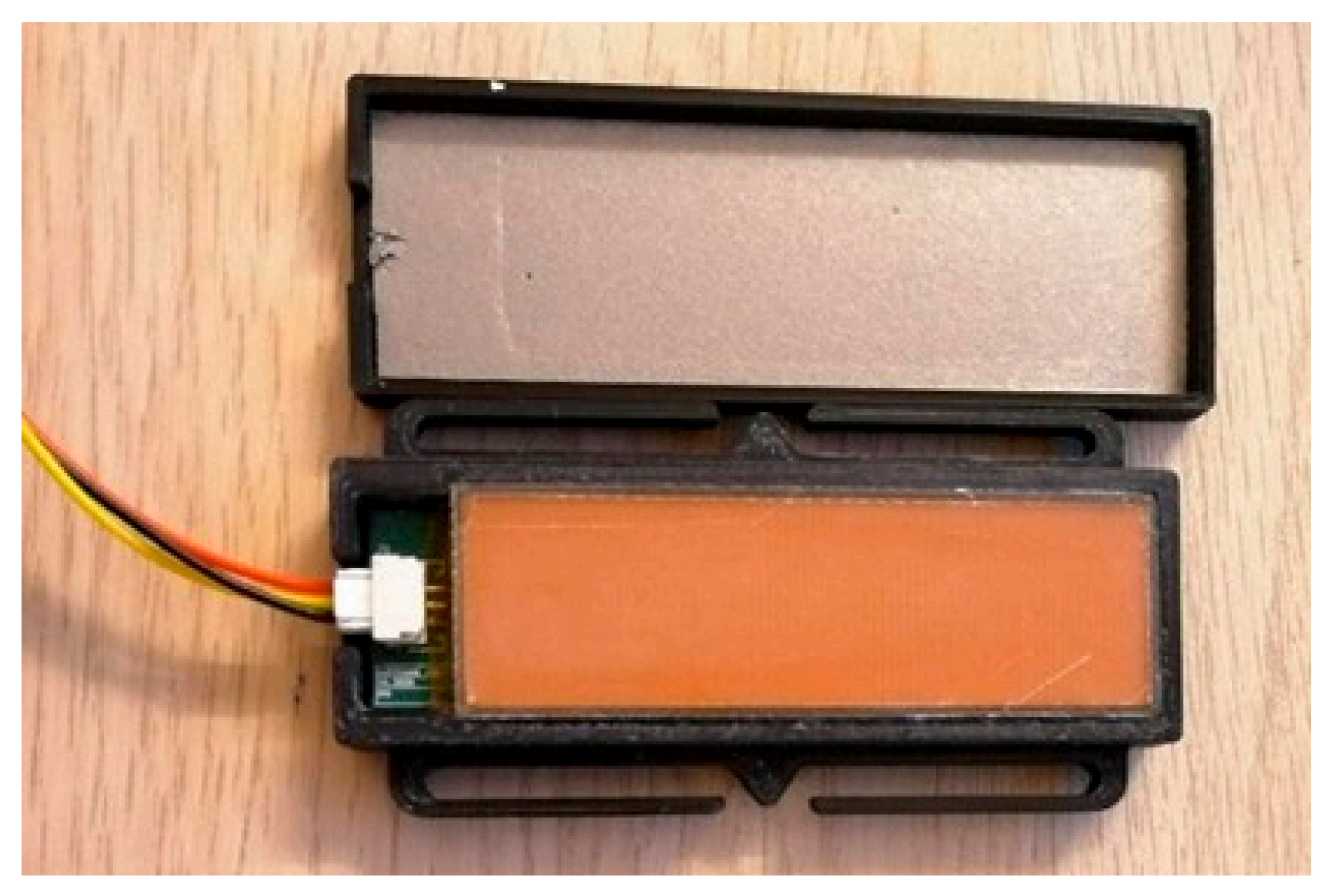
Example of an MW sensor in a housing with shielding.

**Figure 12:**
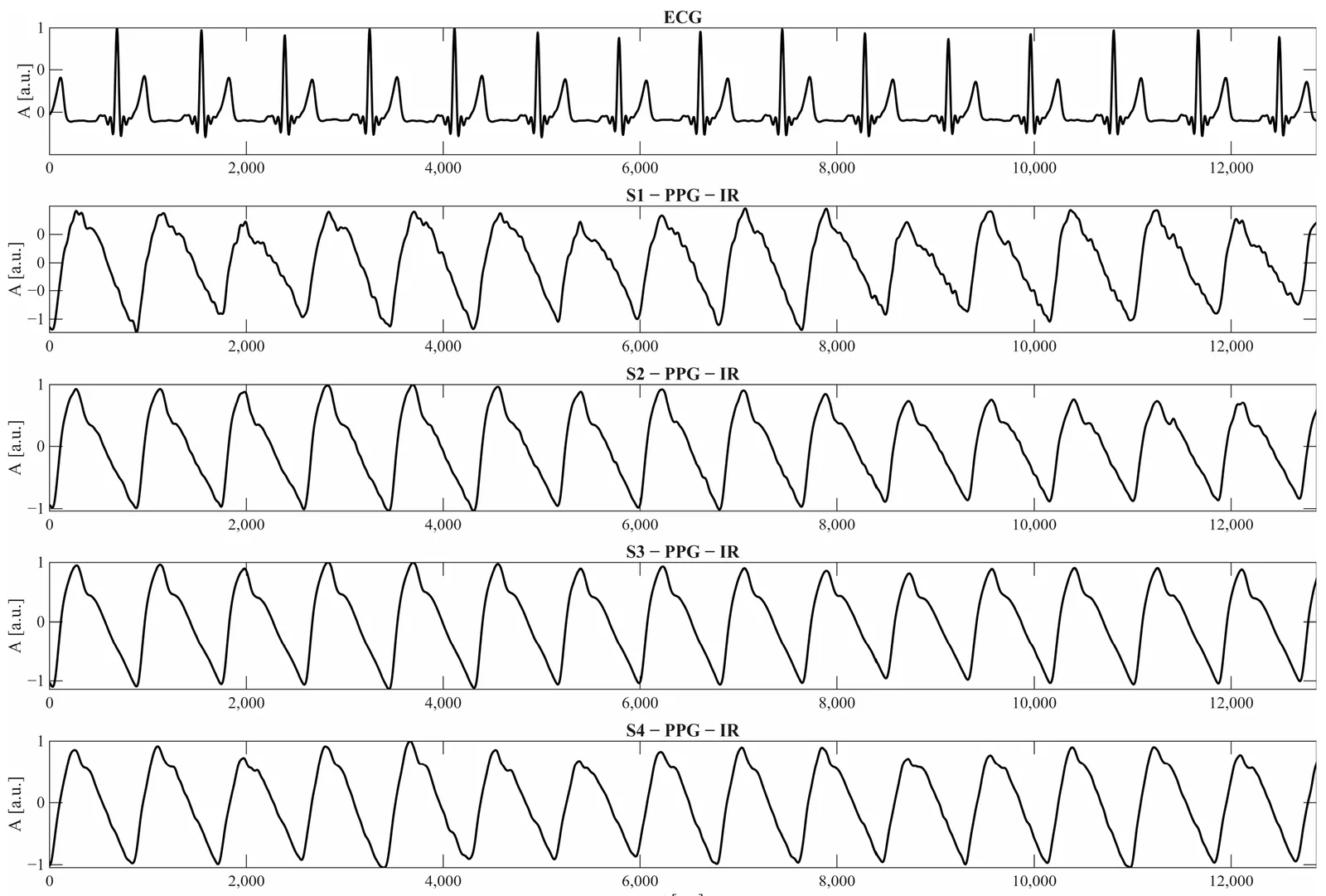
Sample recordings from ECG and PPG sensors.

**Figure 13:**
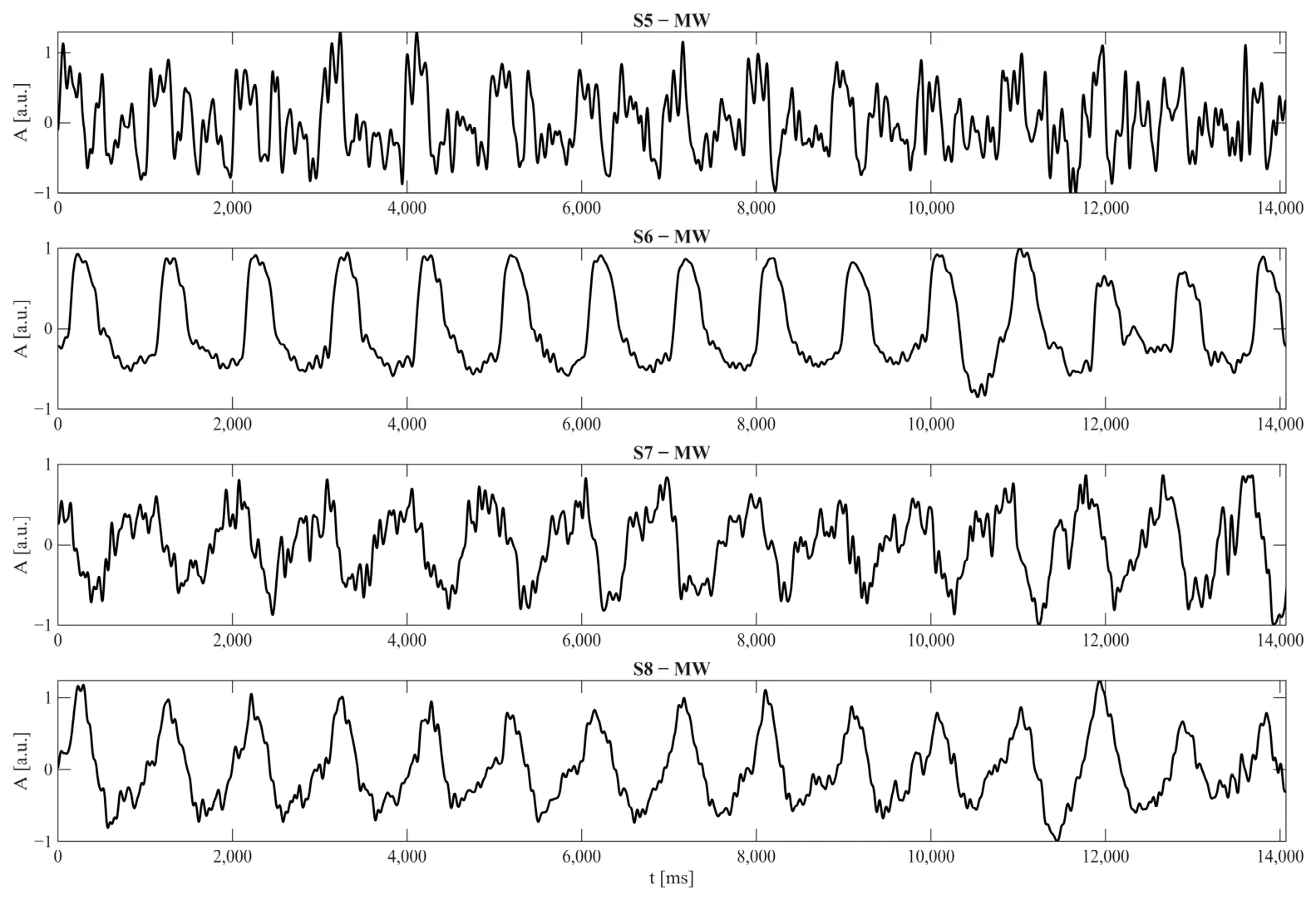
Sample recordings from MW sensors.

**Figure 14:**
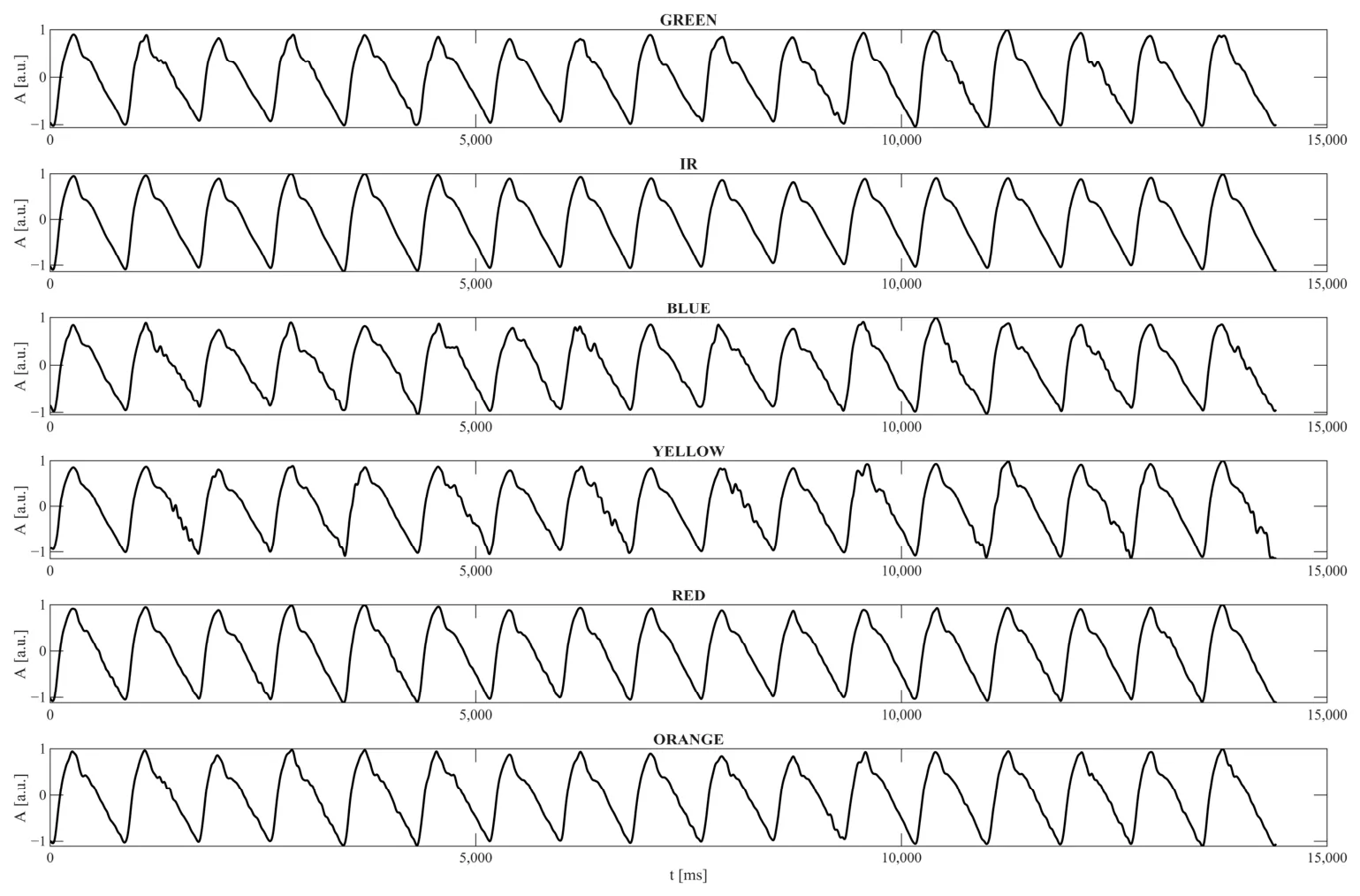
Sample recordings from MWPPG sensor.

**Figure 15:**
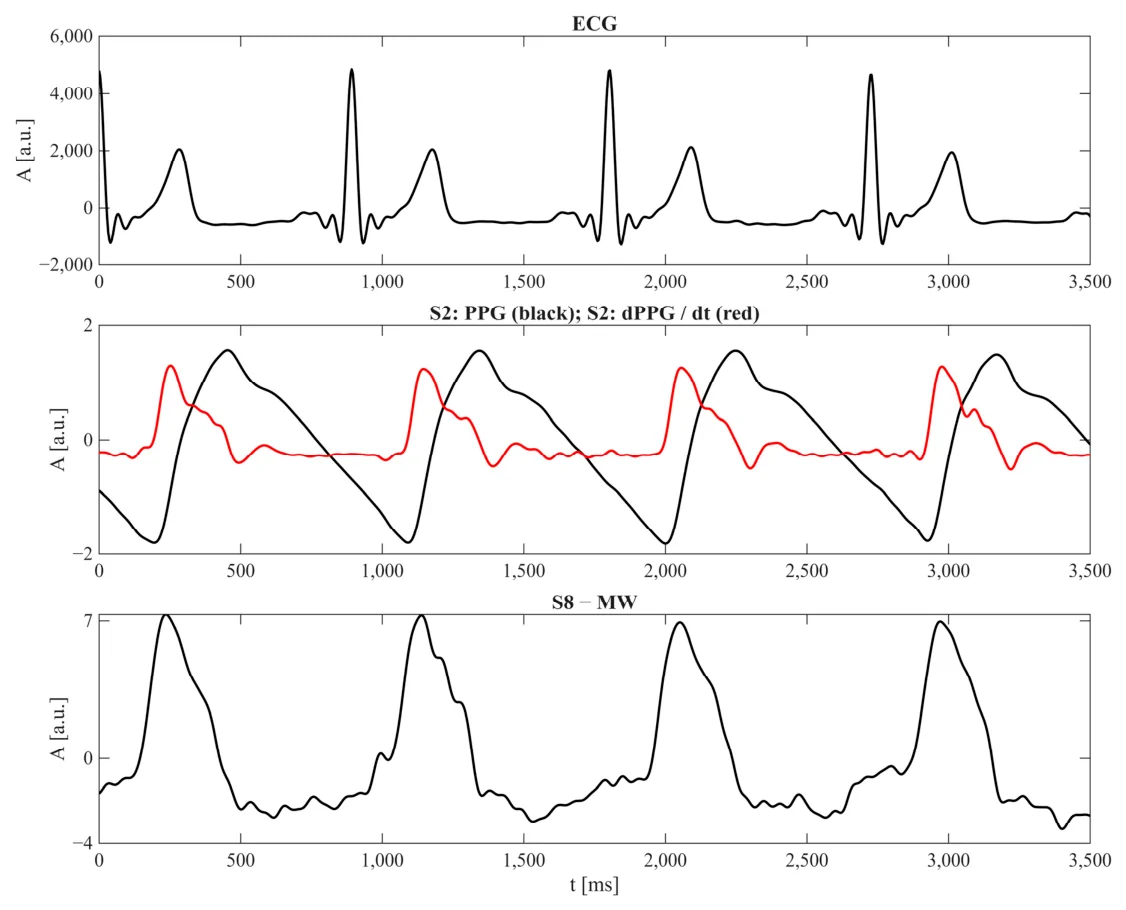
Synchronization of selected biomedical signals measurements, red line represents the first derivative of the PPG.

**Figure 16:**
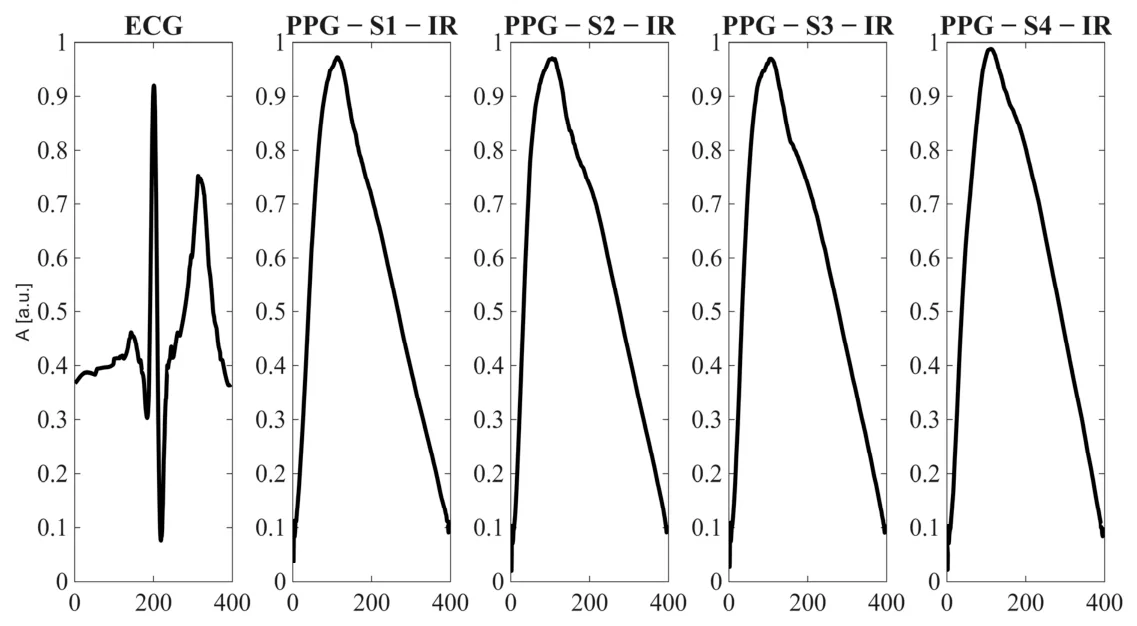
Averaged ECG and PPG pulses of sensors S1–S4.

**Figure 17:**
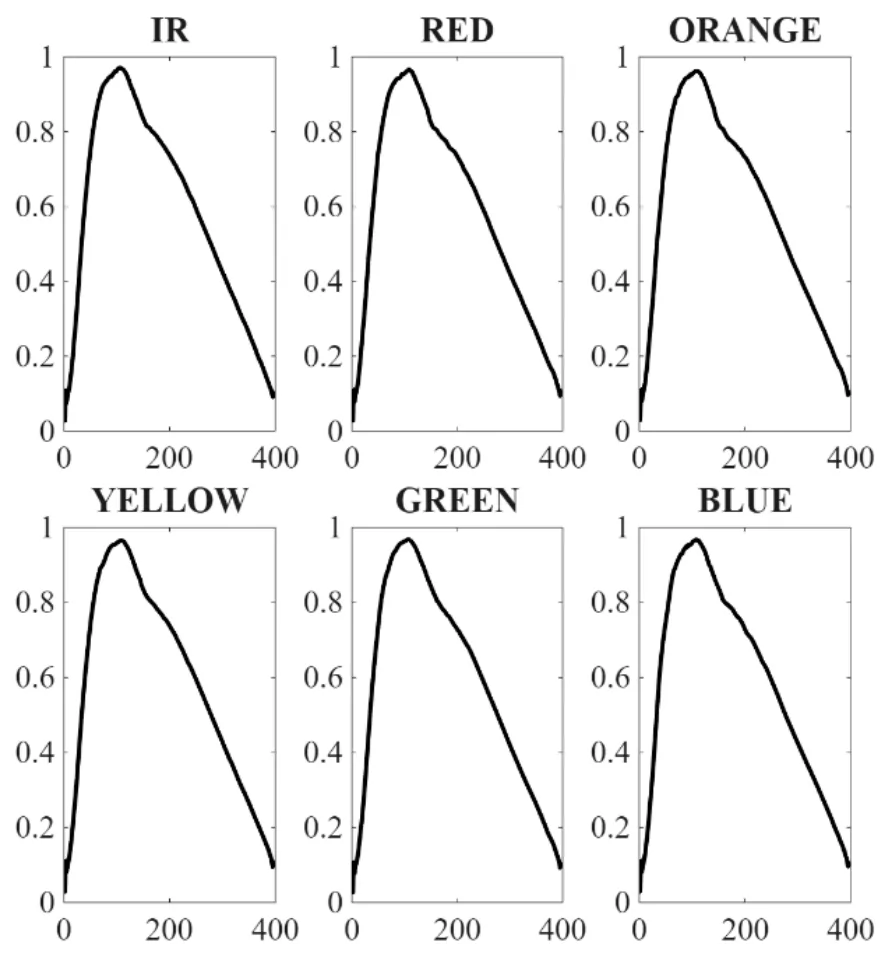
Averaged PPG pulses from the MWPPG sensor.

**Figure 18:**
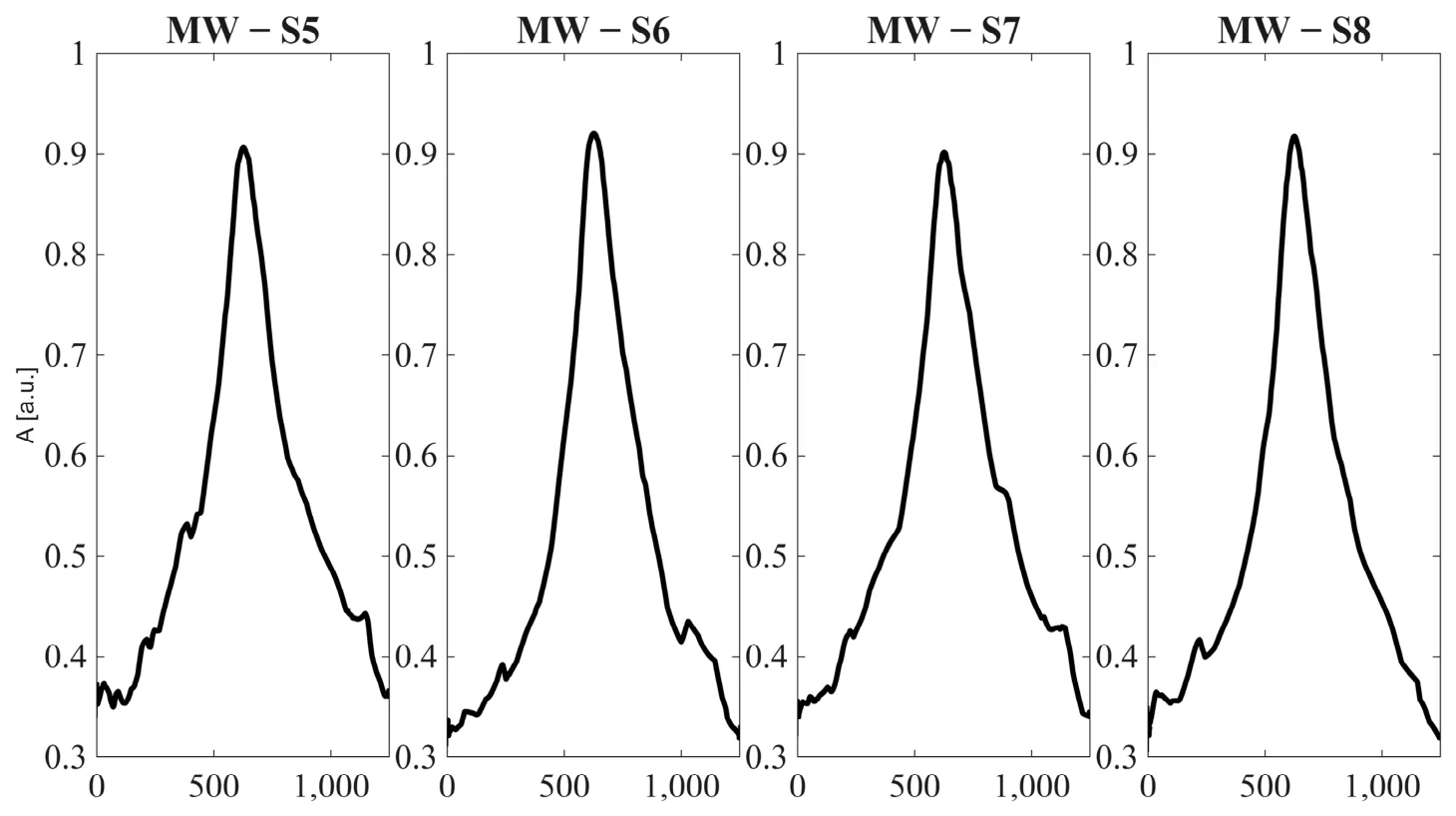
Averaged MW pulses from the S5–S8 sensors.

**Figure 19:**
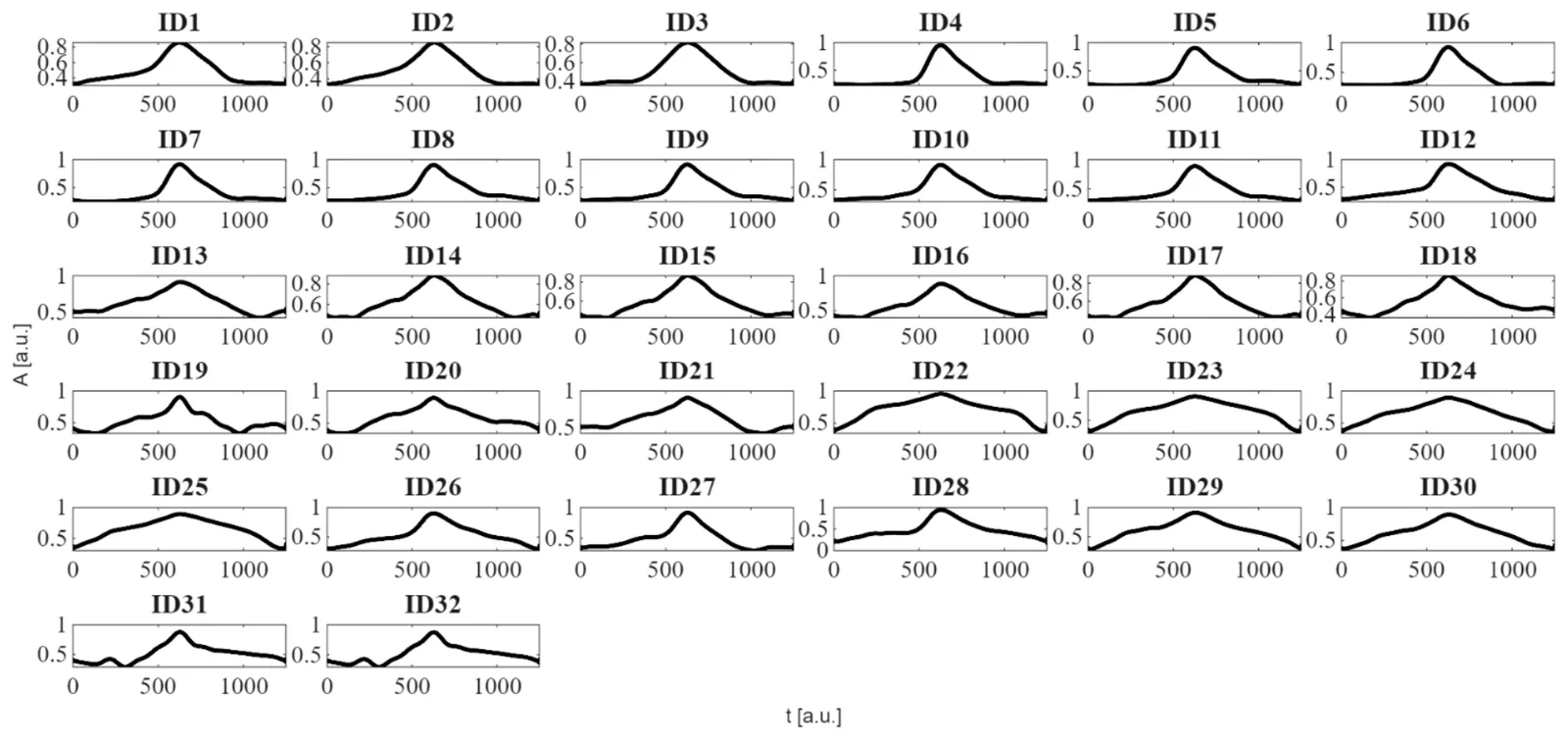
Averaged MW pulses from sensor S6 for each volunteer.

**Figure 20:**
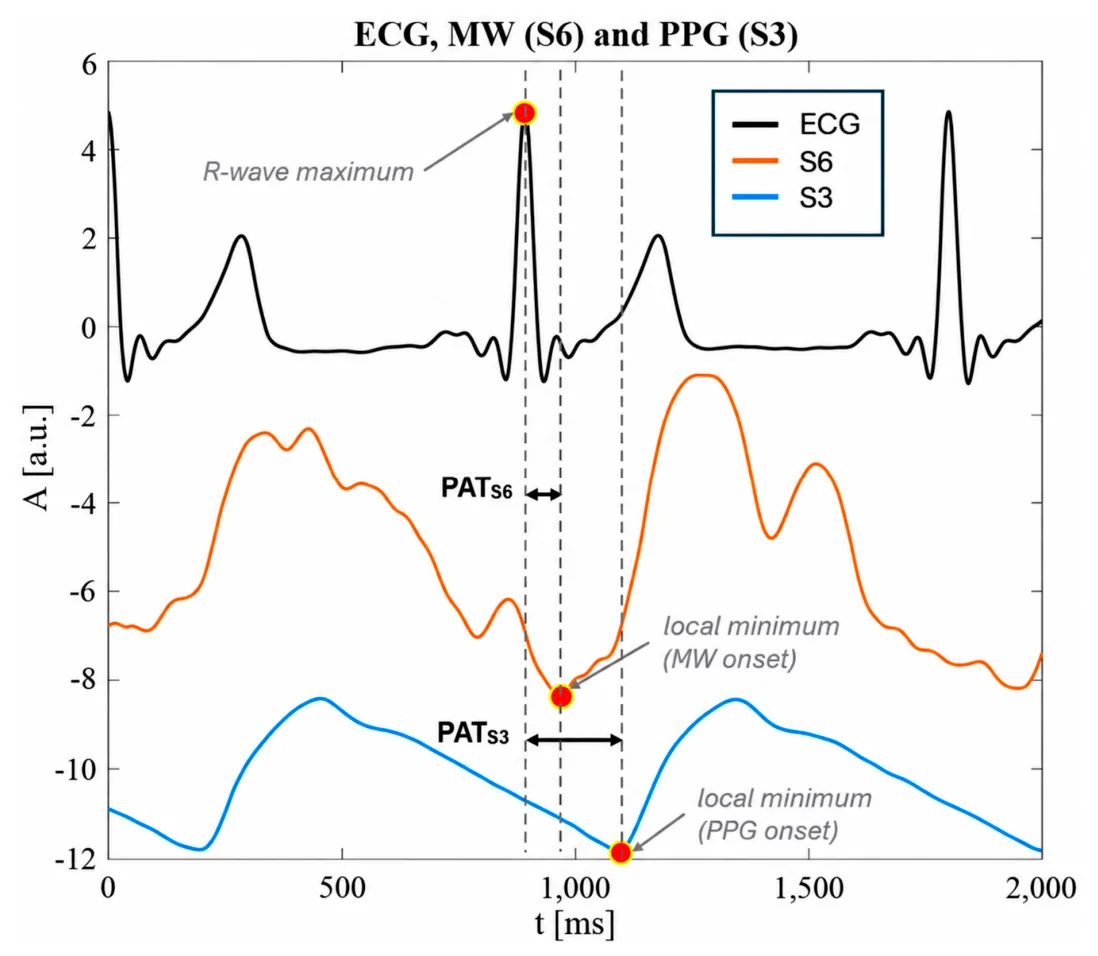
ECG, PPG and MW signals with marked fiducial points used to determine the PAT.

**Figure 21:**
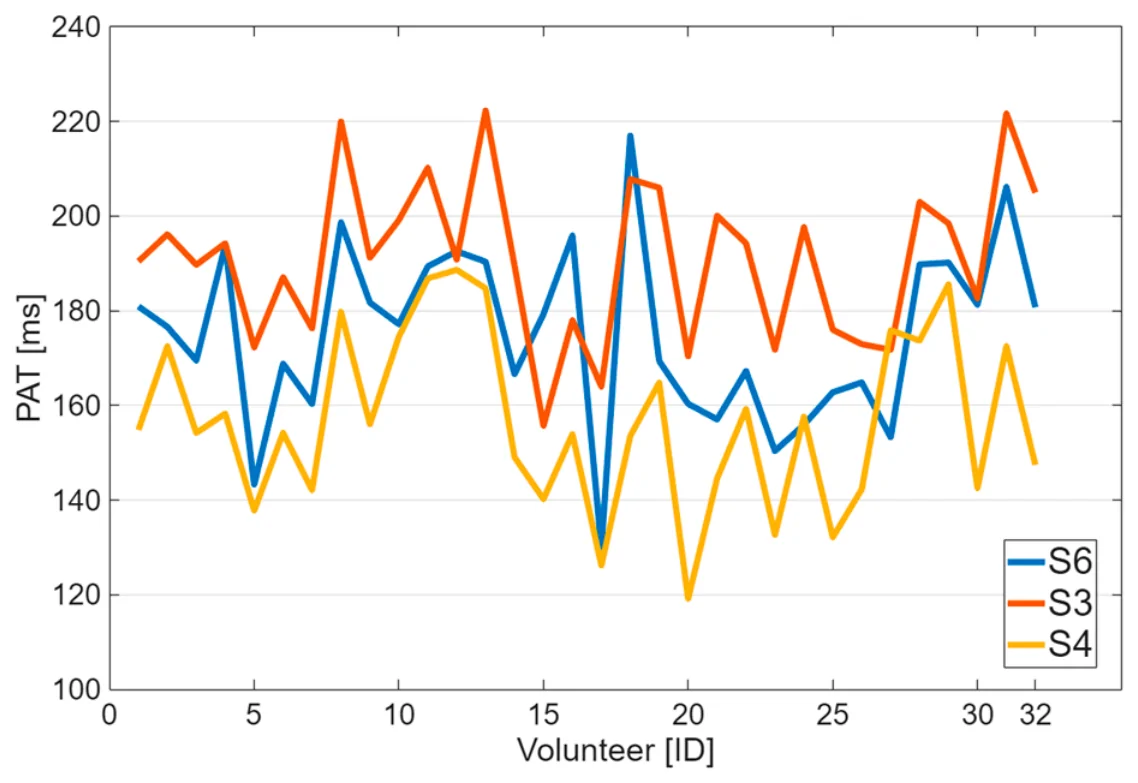
Averaged PAT from sensor S3, S4 and S6 for each volunteer.

**Table 1:** Characteristics of the study group.

Parameter	M ± SD (Min–Max) or Number
Number of subjects	32
Sex: male/female	18/14
Age	45.45 ± 13.56 (21–64)
Height [cm]	173.94 ± 9.08 (152–192)
Weight [kg]	80.58 ± 17.02 (49–120)
Body Mass Index [kg/m^2^]	26.52 ± 4.66 (18–36.44)
Heart Rate [BPm]	71.58 ± 8.70 (55–91)
Waist circumference [cm]	92.03 ± 15.33 (61–125)

M—mean value, SD (standard deviation), min—minimum value, max—maximum value.

**Table 2:** Count of correct and incorrect pulses from individual sensors after signal quality assessment (recording time is about 1 h).

Sensor	S1 (ECG)	S1 (PPG)	S2 (PPG)	S3 (PPG)	S4 (PPG)	S5 (MW)	S6 (MW)	S7 (MW)	S8 (MW)
Count of correct pulses	105,560	26,740	78,911	84,676	39,387	34,672	38,176	26,540	35,692
Count of incorrect pulses	35,022	107,259	55,875	48,451	94,062	94,141	90,954	102,325	92,290
Total	140,582	133,999	134,786	133,127	133,449	128,813	129,130	128,865	127,982

**Table 3:** Signal quality for different sensors and measurement locations; ME ± SD (95% confidence interval).

Sensor	SNR	RSQI	Entropy	NS	Skewness	HR Error	Precision	Recall	F1-Score
S1	8.66 ± 2.17 (7.82–9.50)	0.99 ± 0.00 (0.99–0.99)	5.65 ± 0.09 (5.61–5.69)	0.62 ± 0.07 (0.59–0.65)	−0.23 ± 0.14 (−0.29–0.17)	3.40 ± 3.32 (1.86–4.94)	0.93 ± 0.06 (0.90–0.96)	0.24 ± 0.19 (0.16–0.32)	0.34 ± 0.22 (0.25–0.44)
S2	12.28 ± 2.19 (11.43–13.13)	0.99 ± 0.00 (0.99–0.99)	5.61 ± 0.10 (5.57–5.65)	0.65 ± 0.04 (0.63–0.66)	−0.26 ± 0.09 (−0.30–0.23)	0.85 ± 0.53 (0.63–1.06)	0.91 ± 0.07 (0.88–0.94)	0.67 ± 0.24 (0.58–0.76)	0.74 ± 0.21 (0.66–0.82)
S3	12.19 ± 2.45 (11.24–13.14)	0.99 ± 0.00 (0.99–0.99)	5.70 ± 0.10 (5.66–5.74)	0.63 ± 0.06 (0.61–0.66)	−0.27 ± 0.10 (−0.31–0.24)	0.82 ± 0.58 (0.59–1.06)	0.90 ± 0.08 (0.87–0.93)	0.67 ± 0.21 (0.60–0.75)	0.74 ± 0.17 (0.68–0.81)
S4	12.61 ± 3.98 (11.05–14.27)	0.99 ± 0.00 (0.99–0.99)	5.75 ± 0.08 (5.71–5.78)	0.56 ± 0.06 (0.53–0.59)	−0.30 ± 0.14 (−0.36–0.24)	1.02 ± 0.71 (0.41–2.63)	0.92 ± 0.06 (0.90–0.95)	0.66 ± 0.22 (0.56–0.75)	0.71 ± 0.2 (0.66–0.82)
S5	5.91 ± 0.98 (5.52–6.30)	0.91 ± 0.02 (0.90–0.92)	5.46 ± 0.05 (5.45–5.48)	0.44 ± 0.05 (0.42–0.46)	−0.27 ± 0.31 (−0.40–0.15)	13.82 ± 2.78 (12.66–14.99)	0.90 ± 0.08 (0.87–0.93)	0.21 ± 0.06 (0.19–0.23)	0.33 ± 0.07 (0.31–0.36)
S6	7.11 ± 1.27 (6.60–7.61)	0.93 ± 0.02 (0.93–0.94)	5.37 ± 0.10 (5.33–5.41)	0.41 ± 0.07 (0.38–0.44)	−0.31 ± 0.76 (−0.61–0.01)	16.48 ± 3.36 (15.08–17.88)	0.90 ± 0.08 (0.87–0.93)	0.27 ± 0.14 (0.21–0.32)	0.41 ± 0.16 (0.35–0.47)
S7	7.95 ± 1.26 (7.39–8.40)	0.94 ± 0.02 (0.93–0.94)	5.34 ± 0.11 (5.29–5.38)	0.42 ± 0.10 (0.38–0.46)	−0.14 ± 0.92 (−0.50–0.22)	15.77 ± 3.41 (14.34–17.19)	0.90 ± 0.08 (0.87–0.93)	0.29 ± 0.14 (0.23–0.34)	0.43 ± 0.15 (0.38–0.49)
S8	6.98 ± 1.11 (6.54–7.42)	0.91 ± 0.02 (0.90–0.92)	5.45 ± 0.06 (5.43–5.47)	0.44 ± 0.08 (0.41–0.47)	−0.23 ± 0.31 (−0.35–0.10)	14.11 ± 4.55 (12.21–16.01)	0.90 ± 0.07 (0.87–0.93)	0.25 ± 0.14 (0.19–0.30)	0.36 ± 0.14 (0.30–0.42)

**Table 4:** Summary of other similar works.

Recorded Signals	Sensor Locations	Trials (N, t)	Reference, Year
ECG, PPG, BT, ACC, MIC	chest sensor	Yes (NA, NA)	[36], 2022
ECG, AAAP, ACC	sensor and electrodes in the shirt	NA	[37], 2015
MWPPG	reflective PPG sensor on the wrist, transmissive PPG sensor on the finger	Yes (10, 10 min)	[38], 2023
ECG, PPG, FP	PPG sensor on the finger, thumb for ECG	Yes (20, 1 min)	[39], 2025
VCG (SCG + GCG), ECG, PPG, ICG	ICG electrodes in the neck and chest, ECG electrodes in the chest, PPG sensor in the earlobe, VCG sensor in the chest	Yes (20, 80 min)	[40], 2025
BT, CIm	wearable sensors	NA	[41], 2025
ECG, PPG	chest ECG electrodes, finger PPG sensor	NA	[42], 2022
ECG, BCG, PPG	sensor integrated into the mattress, PPG sensor on the toe	Yes (4, 15 min)	[43], 2024
PPG, IPG, ECG	PPG and IPG sensors on the wrist, ECG electrodes on the chest	Yes (10, 120 min)	[44], 2019
SCG, IP, ECG, PPG	chest sensor	Yes (14, NA)	[45], 2025
ECG, PPG, ACC, GCG	PPG, ECG, and ACC sensors on the wrist, ACC and GYR sensors on the foot	Yes (4, 5 min)	[46], 2025
ECG, PPG, IPG	ECG electrodes in the chest, IPG sensor in the wrist, PPG sensor in the finger	Yes (10, 25 min)	[47], 2022
PPG, IPG	wrist sensor	Yes (10; NA)	[48], 2018
ECG, BCG	flexible patch-type electrode on the wrist	Yes (10, NA)	[49], 2024
PPG, IPG, ECG, ST	ECG electrodes in the abdomen, PPG sensor clip in the index finger	Yes (15, 10 min)	[50], 2023
PPG, ECG, IPG, ST	ECG electrodes in the chest, IPG sensor in the wrist, PPG and ST sensors in the finger	Yes (23, 11 min)	[51], 2025
IPG, PPG	IPG sensor on the wrist over the radial artery, PPG on the index finger	Yes (15, 12 min)	[52], 2019
IPG, PPG, **mmRF**	sensor on the left wrist	NA	[53], 2025
PPG, IPG	sensor on the left wrist	Yes (8, 12 min)	[54], 2024
ECG, PPG, MWPPG, **MW**, ACC	ECG electrodes in the chest; transmissive PPG on the left index finger; reflective PPG on the left wrist and forehead; MWPPG on the left middle finger; MW in the chest, left bicep and left wrist; ACC in each sensors	Yes (32, 60 min)	This work

Body temperature (BT); Ballistocardiography (BCG); Seismocardiography (SCG); Impedance Cardiography (ICG); accelerometer (ACC); Microphone (MIC); Finger Pressure (FP); Camera Images (CIm); Gyroscope (GCG); Abdominal Aortic Aneurysm Pressure (AAAP); Impedance PPG (IPG); skin temperature (ST); millimeter-wave radio frequency (mmRF); microwave (MW); sample size (N); not available (NA); (t) trial duration.

## Data Availability

For this study, we created our own dataset. Because further research is ongoing, we cannot publish this dataset at this time.
